# Association of greenspaces exposure with cardiometabolic risk factors: a systematic review and meta-analysis

**DOI:** 10.1186/s12872-024-03830-1

**Published:** 2024-03-20

**Authors:** Yasaman Sharifi, Sahar Sobhani, Nahid Ramezanghorbani, Moloud Payab, Behnaz Ghoreshi, Shirin Djalalinia, Zahra Nouri Ghonbalani, Mahbube Ebrahimpur, Maysa Eslami, Mostafa Qorbani

**Affiliations:** 1 Department of Radiology, School of Medicine, Iran University of Medical Sciences, Tehran, Iran; 2 Endocrinology and Metabolism Research Center, Endocrinology and Metabolism Clinical Sciences Institute, Tehran University of Medical Sciences, Tehran, Iran; 3 Non-Communicable Diseases Research Center, Alborz University of Medical Sciences, Karaj, Iran; 4 Department of Development and Coordination Scientific Information and Publications, Deputy of Research and Technology, Ministry of Health and Medical Education, Tehran, Iran; 5 Non-Communicable Diseases Research Center, Endocrinology and Metabolism Population Sciences Institute, Tehran University of Medical Sciences, Tehran, Iran; 6 Development of Research & Technology Center, Ministry of Health and Medical Education, Tehran, Iran; 7 Social Determinants of Health Research Center, Alborz University of Medical Sciences, Karaj, Iran; 8 Elderly Health Research Center, Endocrinology and Metabolism Population Sciences Institute, Tehran University of Medical Sciences, Tehran, Iran; 9 Faculty of Medicine, Tehran Medical Sciences, Islamic Azad University, Tehran, Iran

**Keywords:** Cardiometabolic, Greenspace, Hyperglycemia, Natural space, Parks, Blood pressure, Lipid profiles, Urban environment

## Abstract

**Background:**

Cardiometabolic conditions are major contributors to the global burden of disease. An emerging body of evidence has associated access to and surrounding public open spaces (POS) and greenspace with cardiometabolic risk factors, including obesity, body mass index (BMI), hypertension (HTN), blood glucose (BG), and lipid profiles. This systematic review aimed to synthesize this evidence.

**Methods:**

This systematic review was conducted based on the PRISMA guidelines. Four electronic databases including Web of Science, PubMed, Scopus, and Google Scholar were searched for eligible articles published until July 2023. All observational studies which assessed the association of greenspace and POS with cardiometabolic risk factors including obesity, BMI, HTN, BG, and lipid profiles were included and reviewed by two authors independently. Heterogeneity between studies was assessed using the I^2^ index and Cochrane’s Q test. Random/fixed effect meta-analyses were used to combine the association between greenspace exposure with cardiometabolic risk factors.

**Results:**

Overall, 118 relevant articles were included in our review. The majority of the articles were conducted in North America or Europe. In qualitative synthesis, access or proximity to greenspaces or POS impacts BMI and blood pressure or HTN, BG, and lipid profiles via various mechanisms. According to the random effect meta-analysis, more access to greenspace was significantly associated with lower odds of HTN (odds ratio (OR): 0.81, 95% confidence intervals (CIs): 0.61–0.99), obesity (OR: 0.83, 95% CIs: 0.77–0.90), and diabetes (OR:0.79, 95% CI: 0.67,0.90).

**Conclusions:**

Findings of this systematic review and meta-analysis suggested that greenspace accessibility is associated with some cardiometabolic risk factors. Improving greenspace accessibility could be considered as one of the main strategies to reduce cardiometabolic risk factors at population level.

**Supplementary Information:**

The online version contains supplementary material available at 10.1186/s12872-024-03830-1.

## Introduction

Cardiometabolic risk factors (CMRFs) including obesity, hypertension, dysglycaemia, and dyslipidemia are among the main risk factors based on the latest global burden of disease report. Resulting in tremendous expenses and a significant amount of morbidity and mortality worldwide. Thus preventive measures to reduce the imposing threats of CMRFs are highly desirable [1].

Greenspace is hypothesized to improve cardiometabolic health by increasing physical activity, reducing stress, and minimizing exposure to air pollution and noise [2]. Greenspaces are usually defined as a land that is partly or completely covered with grass, trees, shrubs, or other vegetation); such as parks, community gardens, and cemeteries [3].

According to World Health Organization (WHO) reports, in 2016, about 39% of adults over 18 years of age (39% men and 40% women) were reported as overweight [4]. Obesity, as we know, is caused by the imbalance of energy intake and the amount of energy consumed through basic metabolic processes and physical activities [4]. Since pharmacological or surgical treatments for obesity are commonly pricey, complicated, and inaccessible to all patients and they are also not lasting solutions. recent research has focused on environmental risk factors that have contributed to obesity and the modulation of these risk factors [5–8]. According to a recent review of the evidence supporting a link between access to greenspace and weight, nearly 70% of studies found a positive or weak association between greenspace and obesity-related health indicators [9].

The next leading cause of CVDs is hypertension (HTN) [7]. Community-level behavioral interventions are suggested to be important tools for controlling HTN at the population level [10, 11]. In recent years, an increasing number of epidemiological studies have looked into the link between greenspace and BP [10, 12–18]. While some studies have reported that more greenspace is associated with lower BP [12], others have been inconclusive [19–21].

Dyslipidemia (abnormalities in blood lipids) is another major risk factor for atherosclerotic cardiovascular disease [22]. Dyslipidemia is a global problem and continues to rise in prevalence [23]. Previous studies have shown that higher exposure to greenspaces is likely to reduce the risk of dyslipidemia [23–25]. Some epidemiological studies have also looked into the relationship between greenness and blood lipids, but the results have been inconsistent [26, 27].

Type 2 diabetes (T2DM) is another CMRF. In most studies regarding T2DM, most of the attention has been given to individual risk factors such as social determinants [28], health-related behaviors [29], and biological attitudes [30], with little attention paid to the role of the residential environment [31]. Previous research has found a link between the abundance of residential greenspace and a lower risk of T2DM [32–37].

It has been previously examined whether greenspace and CMRFs of CVDs are linked independently or simultaneously in a single population or as a systematic review evaluating one of these risk factors [9, 38–42]. To the best of our knowledge, no systematic review has addressed the relationship between greenspace and all of the mentioned CMRFs simultaneously and thoroughly (for example the association between HTN, BG, or lipid profile and greenspace were not evaluated in a systematic review article; hence, an adequate review of recent studies on these CMRFs would be valuable to determine the prognostic effect of greenspace on them as well as CVDs.

Hence, we aimed to systematically review and synthesize the available evidence on the associations between greenspace and CMRFs including obesity, hypertension, diabetes, and dyslipidemia. Moreover, all types of variables (continuous and qualitative) were included in the systematic review.

## Materials and methods

**Fig. 1:**
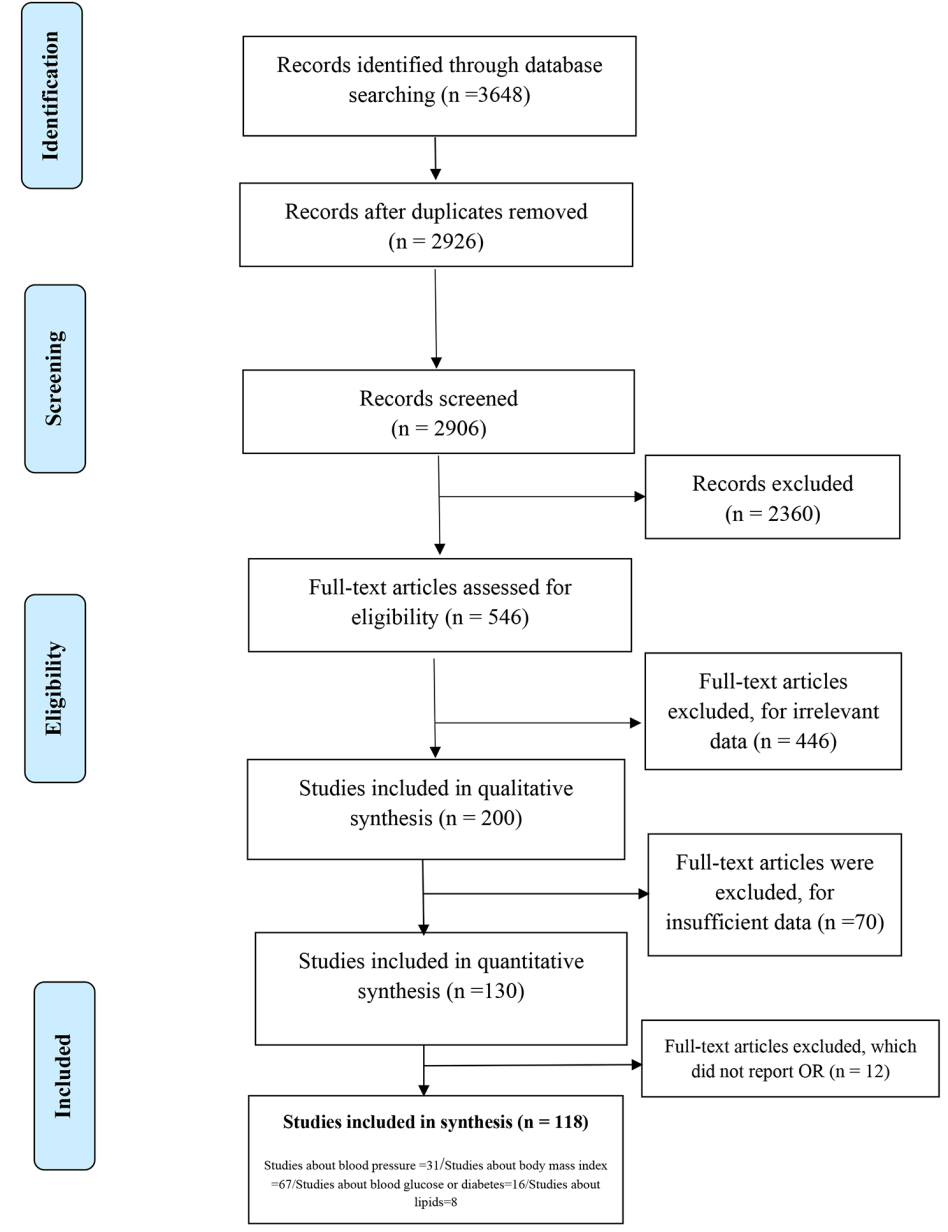
PRISMA diagram for selection of primary studies about CMRFs and greenspaces

We conducted our systematic review and meta-analyses based on the Preferred Reporting Items for Systematic revReviewsd Meta-analyses (PRISMA) guidelines [43, 44] and all steps were followed according to a predefined protocol. We searched PubMed/MEDLINE, Web of Sciences (ISI), Scopus, and Google Scholar for articles published until July 2023. The main root of search strategies developed based on Exposure to greenspace either by “Proximity”, or “Accessibility” to “greenspace” with CMRFs as continuous (BMI, WC, BP, BG, TG, LDL, HDL, Cholesterol) and categorical outcomes (“obesity, HTN, “diabetes”, “high total cholesterol”, “high triglyceride”, “high LDL, low HDL, dyslipidemia)” (Fig. 1and Table 1). The reference list of relevant articles was reviewed as well to retrieve further eligible studies that were not found through our search.

**Table 1:** General characteristics of included studies

No	Author (Year)	Country	Urban or Rural?	Type of Study	Sampling	Sample size	Dependent Variables	Age	Quality Assessment
1	Zhang (2019) [74]	USA	Urban	Cohort	census	9521	BMI	20–60	8/10
2	Vaccaro (2019) [68].	USA	-	cross-sectional	Random	42,828	BMI	10–17	7/9
3	Astell-Burt (2014) [136]	Australia	-	cross-sectional	Random	246, 920	BMI	45–106 45 ≤	7/9
4	U. Goldsby (2016) [55]	USA	Urban	Cohort	A convenience samples	1443	BMI	< 19	9/10
5	Alexander(2013) [50]	USA	-	cross-sectional	Random-digit-dialing	44 278	Obesity	6–17	7/9
6	Bai(2013) [51]	USA	-	cross-sectional	Census	3,906	BMI	18–63	6/9
7	Bell(2008) [52]	USA	Urban	cohort	Census	3831	BMI	3–16	8/10
8	Bird(2016) [76]	Canada	Urban	Cohort	Census	380	Obesity	8–10	9/10
9	Burgoine(2015) [91]	UK	Urban	cross-sectional	Base line data	94	BMI	5–11	7/9
10	Dadvand(2014) [105]	Spain	Urban	cross-sectional	Random	3,178	sedentary behavior, obesity, asthma, and allergy	9–12	8/9
11	Davidson(2010) [77]	Canada	Urban & Rural (Urban = 47.2 Town = 16.1 Rural = 36.7)	-	Random	148	Body weights	Students grade 5	-
12	Gose(2013) [98]	Germany	-	-	Data were collected as part of the Kiel Obesity Prevention Study	485	weight status	age at baseline: 6.1 (5.8–6.4)	-
13	Assis (2018) [145]	Brazil	-	cross-sectional	Census	408	Overweight	6–15	6/9
14	Hobbs(2018) [92]	UK	Urban & Rural (Urban = 88.60 Rural = 11.40)	cross-sectional	Yorkshire Health Study	22,889	BMI	18–86	8/9
15	Klompmaker(2018) [99]	Dutch	Urban & Rural (very highly urbanized = 15 highly urbanized = 25 moderately urbanized = 18 low urbanized = 23 not urbanized = 19)	cross-sectional	using data from a Dutch national health survey	387,195	Overweight	≥ 19	7/9
16	Lovasi(2011) [61]	USA	Urban	cross-sectional	Census	428	Adiposity	2–5	7/9
17	Manandhar(2019) [125]	Nepal	Urban	cross-sectional	Random	440	Overweight/ Obesity	6–13	7/9
18	Mathis(2017) [62]	USA	Urban	cross-sectional	Census	217	BMI	65+	6/9
19	Mena(2014) [146]	Chile	Urban	cross-sectional	Random	832	BMI	18–74	7/9
20	Mendes(2013) [147]	Brazil	Urban	cross-sectional	Random	3404	Overweight	18 ≤	6/9
21	Hughey(2017) [162]	USA	-	cross-sectional	Census	13,469	Obesity	3rd–5th grade	7/9
22	Grit Müller (2018) [100]	Germany	Urban	cross-sectional	Census	1312	BMI/T2DM	25–74	7/9
23	Nicolle-Mir (2018) [107]	Lithuania.	Unknown	cross-sectional	Data used from cohort study	1489	Overweight	4–6	8/9
24	Nies(2015) [79]	Idaho	Urban& Rural	Retrospective base EHR	Census	9800	Obesity	18–105	7/10
25	Picavet(2016) [90]	Netherlands	Urban &Rural	cross-sectional and longitudinal	Random	4005	Height, weight, blood pressure	20–59	8/9
26	Potestio (2009) [80]	Canada	Urban	cross-sectional	Census	6,772	Overweight/Obesity	3–8	8/9
27	Potwarka(2008) [81]	Canada	-	-	Random	108	Weight Status	2–17	-
28	Putrik(2015) [102]	Netherlands	-	cross-sectional	-	9771	Overweight/Obesity	18–65	7/9
29	Rossi(2018) [149]	Brazil	-	cross-sectional	Random	2,152	BMI	7–14	8/9
30	Rundle(2013) [65]	USA	-	cross-sectional	Census	13,102	BMI	-	8/9
31	Schüle(2016) [101]	Germany	-	cross-sectional	Census	3499	Overweight	5–7	6/9
32	Singh(2010) [66]	USA	-	cross-sectional	Random	44,101	Obesity	10–17	7/9
33	Sullivan(2014) [67]	USA	-	cross-sectional	Census	6082	Obesity	≥ 18	7/9
34	Toftager(2011) [103]	Denmark	Urban	cross-sectional	Census	14,566	Physical activity	≥ 16	6/9
35	van der Zwaard(2018) [93]	UK	-	Cohort	-	6001	BMI	3–11	8/10
36	Veitch(2016) [69]	Australia	Urban & Rural, 40 Urban area)	cross-sectional	Random	1848	Overweight and Obesity	33.5- 50.0	7/9
		USA				489	Overweight and Obesity	43.7- 57.1	7/9
37	Velásquez-Meléndez(2013) [150]	Brazil	Urban	cross-sectional	Random	3,425	Overweight and Obesity	18–65	8/9
38	Veugelers (2008) [82]	Canada	Urban &Rural (Urban = 55 and Rural = 45)	-	-	5 200	Diet, Physical activity and Overweight	10–11	-
39	Wall(2012) [70]	USA	Urban/ suburban	cross-sectional	-	2682	Obesity	Mean age 14.5	7/9
40	Wen(2012) [71]	USA	Urban	cross-sectional	census	2000	Obesity	20–64	7/9
41	Wolch(2011) [72]	USA	Urban	cohort	Random	3173	Obesity	9–10	7/10
42	Yang(2018) [73]	USA	Urban	cross-sectional	census	41,283	Overweight and Obesity	3–18	7/9
43	Nesbit(2014) [63]	USA	-	cross-sectional	Random	39,542	Obesity	11 to 17	7/9
44	Pereira(2018) [106]	Portugal	Urban	-	Census	929	Obesity	Mean (± SD) 7.28 (± 1.94)	-
45	Akpinar(2017) [126]	Turkey	Urban	Cross-sectional	Census	422	Physical activity/Screen time/General health/Overweight	Children 1–18	7/9
46	Benjamin-Neelon (2019) [75]	Mexico	-	Cohort	Random	102	BMI	children 3–5	9/10
47	Bjork (2008) [108]	Sweden	Urban	Cross-sectional	Census	24,819	Physical activity, Obesity and Wellbeing	18–80	7/9
48	Brown(2009) [53]	USA	-	Cross-sectional	Census	5000	BMI, overweight, and obesity	25–64	7/9
49	Browning and Rigolon (2018) [54]	USA	Urban	Cross-sectional	Census	97,574,613	Obesity and Mental health	> 18	8/9
50	Coombes (2010) [95]	UK	Urban	Cross-sectional	Census	6821	Physical activity and Overweight	> 16	7/9
51	Cummins and Fagg (2012) [96]	UK	Urban &Rural (Urban = 80.3, Town = 9.5, village = 10.2)	Cross-sectional	Random	79,136	Weight status	> 18	7/9
52	Dempsey(2018) [109]	Ireland	Urban	Cross-sectional	Census	8175	Obesity	≥ 50	6/9
53	Ellaway (2005) [97]	UK	-	Cross-sectional	Census	6919	Obesity	Adults age not mentioned	8/9
54	Feng (2018) [138]	Australia	Urban &Rural (Urban = 12.9, regional = 11.1 and Rural = 12.2)	Cohort	Census	3843	Obesity	Mothers age not mentioned	7/10
55	Hoehner (2012) [56]	USA	-	Cross-sectional	Random	8857	Cardiorespiratory fitness	20–88	7/9
56	James (2017) [57]	USA	Urban &Rural (Urban = 98.8 Small town& Rural = 1.1)	Cross-sectional	Random	23,435	BMI	60–87	7/9
57	Li (2008) [58]	USA	Urban	Cross-sectional	Random	1221	Adiposity, and Physical activity	50–75	7/9
58	Li (2018) [59]	USA	Urban	Cross-sectional	-	149,797	BMI	18–84	7/9
59	Liu (2007) [60]	USA	Urban &Rural	Cross-sectional	Census	7334	Overweight	Children 3–18	7/9
60	Mowafi (2012) [127]	Egypt	Urban	Cross-sectional	-	3546	BMI	≥ 22	6/9
61	Nielsen and Hansen (2007) [104]	Denmark	Urban	Cross-sectional	Random	2000	Obesity, Overweight, Mental stress, Physical activity	18–80	7/9
62	Norman (2006) [64]	USA	-	Cross-sectional	-	789	Physical activity and BMI	Children 11–15	7/9
63	Oreskovic (2009) [49]	USA	Urban &Rural	Cross-sectional	Census	21,008	Obesity	Children 2–18	7/9
64	Ortega Hinojosa (2018) [48]	USA	-	Cross-sectional	-	5,265,265	Obesity	Children	8/9
65	Pearson (2014) [140]	New Zealand	Urban &Rural	Cross-sectional	Random	12,488	Obesity	> 15	7/9
66	Pereira (2013) [141]	Australia	-	Cross-sectional	Census	10,208	Weight status	> 16	8/9
67	Li(2022) [177]	China	Rural	cross-sectional	Census	8377	Obesity	≥ 18	7/9
68	Abbasi(2020) [15]	Iran	-	cross-sectional	cluster sampling	12,340	Blood Pressure	7–18	7/9
69	Thomas Astell-Burt(2016) [137]	Sydney, Australia.	-	-	Census	7272	Physical activity, Mental and Cardiometabolic health	45 ≤	-
70	Regina Grazuleviciene(2014) [88]	Lithuania	Urban	cross-sectional study	-	3,416 females	Blood Pressure	20–45	6/9
71	Sérgio Rodrigues Moreira(2013) [148]	Pernambuco State, Brazil.	Urban	-	-	500 adults	stress, high blood pressure, and high blood glucose	> 18	-
72	Iana Markevych(2014) [178]	Munich, Germany	-	cross-sectional study	-	2,078 children	Blood Pressure	10 years	7/9
73	Usama Bilal(2016) [179]	Spain	Urban	exploratory study	census	16,000 Electronic health records	Diabetes or Hypertension, Dyslipidemia, Obesity or smoking	≥ 45	7/9
74	H SusanJ Picavet(2016) [111]	Netherlands	Urban &Rural	prospective longitudinal study	an age-sex stratified sample	12,439	BMI/Diabetes/Blood pressure/CVD/Mental health	20–59	6/10
75	Bo-Yi Yang(2019) [14]	China	Urban	Cohort	randomly	24,845 adults	Blood pressure	18–74	8/10
76	Scott C. Brown(2016) [26]	USA	Urban &suburban	retrospective cohort	Census	249,405 Medicare beneficiaries	Diabetes/ Hypertension/ Hyperlipidemia	≥ 65	8/10
77	Catherine Paquet(2014) [139]	Australia	Urban	longitudinal biomedical cohort	randomly	3145 adults	(pre)Diabetes/ Hypertension/ Dyslipidemia/ Abdominal Obesity	≥ 18	7/10
78	Angel M. Dzhambov(2018) [16]	Austria	Urban &Rural	cross-sectional	randomly	555adults	Blood pressure	35–81	6/9
79	Esmée M Bijnens(2017) [12]	Belgium	-	Prospective Cohort	-	278 twins	Blood pressure	18–25	7/9
80	Jie Jiang(2020) [13]	China	Rural	cross-sectional study	-	39,259	Blood pressure	18–79	7/9
81	Marcia P. Jimenez(2020) [17]	New England	-	longitudinal study	random intercepts for each family and for each census tract	517	Blood pressure/BMI	birth (mean age = 1.60 months) childhood (mean age = 7.08 years) adulthood (mean age = 44.41 years)	7/9
82	Ray Yeager(2018) [47]	University of Louisville	Urban &Rural	cross-sectional	-	408	Hypertension/ Hyperlipidemia/ Diabetes mellitus/ Current smoker	51.4 ± 10.8	8/9
83	Li(2022) [129]	China	-	cross-sectional	randomly	8383	Blood pressure	≥ 18	7/9
84	Ruijia Li (2021) [130]	China	Rural	Cohort	-	39,019	Type 2 diabetes mellitus	18–79	8/10
85	Annie Doubleday(2022) [83]	USA	Urban	prospective cohort	-	6814	Type 2 diabetes mellitus	45–84 years	9/10
86	LucíaRodriguez-Loureiro(2022) [180]	Belgium	Urban	longitudinal study	census	2,309,236	Diabetes mortality	40–79 years	7/9
87	Jiaqiang Liao(2019) [131]	China	Urban	Cohort	-	6807	Blood glucose levels	≤ 24 ≥ 35	8/10
88	Anna Ponjoan(2022) [113]	Spain	Urban	retrospective cohort	-	41,463	Myocardial infarction in the population with diabetes	mean68.8 years	8/9
89	Roland Ngom(2016) [152]	Canada	Urban	cross-sectional	census	3,920,000	Cardiovascular morbidity and Diabetes	≥ 20	7/9
90	Soumya Mazumdar(2021) [144]	Australia	Urban	cohort	randomly	267,153	Type 2 Diabetes	≥ 45	7/10
91	Charlotte Clark(2017) [84]	Canada	Urban &Rural	cohort	-	380,738	Diabetes	45–85	8/10
92	Danielle H Bodicoat(2014) [114]	UK	Urban &Rural (Urban = 83.6 Rural = 16.4)	cross-sectional	random	10 476	Type 2 Diabetes	20–75	7/9
93	Alice M. Dalton(2016) [115]	UK	Urban &Rural (Urban = 46.9 Town and fringe = 20.7 Village = 23.6 Hamlet = 8.9)	cross sectional	-	23,865	Diabetes	39.5 -79.1	7/9
94	Shanley Chong (2019) [143]	Australia	Urban	Prospective cohort	random	60 404	Type 2 Diabetes	≥ 45	8/9
95	Thomas Astell-Burt(2014) [32]	Australia	Urban &Rural	-	random	267,072	Type 2 Diabetes	≥ 45	-
96	H.Lee(2015) [181]	Korea	Urban &suburban	Cross-sectional	-	16,178	Physical activity/ Hypertension and Diabetes	Mean age 47.50 ± 12.87	7/9
97	Dadvand (2018) [35]	Iran	Urban &Rural	Population base	Clustering	3844	Blood glucose	7–18	8/9
98	Shujun Fan (2020) [23]	China	Rural	cross- sectional	stratified cluster random	4735	Blood lipids	≥ 18	8/9
99	Hye-Jin Kim (2016) [27]	Korean	-	cross-sectional	-	212,584	Hyperlipidemia	≥ 20	7/9
100	Hari S. Iyer(2020) [132]	Africa	Suburban &Urban&Rural	cross-sectional	-	1178	BMI/ diabetes/ hypertension/ cholesterol	Mean age 46.7	6/9
101	Jie Jiang(2021) [116]	China	Rural	cohort	multi-stage sampling	39,057	Dyslipidemia	mean age 55.6	7/10
102	Iana Markevych(2016) [133]	Germany	Urban	cohort	-	1,552	Blood lipids	10 and 15 years of age	7/10
103	Bo-Yi Yang(2019) [151]	China	Urban	cohort	four- stage cluster random	15,477	Blood lipids	Mean age 44.97	9/10
104	Aliyas et al. (2018) [135]	Iran	Urban	Cross-sectional	random	978	Hypertension	≥ 65	8/9
105	Bauwelinck et al. (2020) [124]	Spain Belgian	Urban	Cross-sectional	random	Barcelona (*n* = 3400) Brussels (*n* = 2335)	Hypertension	≥ 15	7/9
106	Bloemsma et al. (2019) [20]	Holland	Urban & Rural	Cohort	census	2302	Hypertension	Adolescents aged 12 and 16	8/10
107	Braziene et al. (2019) [123]	Lithuania	Urban & suburban	Cohort	Census	739	Hypertension	35–64	8/10
108	de Keijzer et al. (2019) [122]	UK	Urban &Rural	Cohort	-	6076	Metabolic syndrome	45–69	9/10
109	Jendrossek et al. (2017) [21]	Germany	Urban &Rural	Cross-sectional	randomly	Wesel (*n* = 1310) Munich (*n* = 1753)	Hypertension	-	7/9
110	Leng et al. (2020) [134]	China	Urban	Cross-sectional	-	4155	Hypertension(cardiovascular health)	≥ 60	8/9
111	Madhloum et al. (2019) [121]	Belgium	-	Cohort	-	769	Hypertension	Newborns	8/10
112	Plans et al. (2019) [120]	Spain	Urban	Cross-sectional	census	1625	Hypertension (Cardiovascular Risk Factors)	40–75	7/9
113	Poulsen et al. (2021) [87]	USA	Urban &Rural	Cross-sectional	-	9593	Blood Pressure	≥ 18	7/9
114	Ribeiro et al. (2019) [119]	Portugal	Urban &Rural (Predominantly urban = 97.4 Moderately urban = 2.4 Predominantly rural = 0.3)	Cross-sectional	random	3108	Hypertension	7-year-old children	8/9
115	Riggs et al. (2021) [86]	USA	-	Cross-sectional	-	73	Hypertension(cardiovascular disease risk)	23–84	6/9
116	Sarkar et al. (2018) [118]	UK	Urban	Cohort	random	429,334	Hypertension	38–73	8/10
117	Tamosiunas et al. (2014) [117]	Lithuania	Urban	Cohort	random	5112	Hypertension(cardiovascular health)	45–72	8/10
118	Ulmer et al. (2016) [85]	USA	Urban	Cross-sectional study	-	4820	Hypertension	-	7/9

### Inclusion and exclusion criteria

We included all observational studies that (1) assessed the link between greenspace with CMRFs such as overweight or obesity; blood pressure or HTN; BG or diabetes; lipid profiles or dyslipidemia. regardless of time, language, methodology, date of publication, and target groups;(2) assessed greenspace exposure using an objective measure (e.g., normalized difference vegetation index [NDVI], a vegetation index that assesses chlorophyll calculated as the ratio of near-infrared minus red light divided by near-infrared plus red light and measured distance to the nearest greenspace) or subjective measure (e.g., self-reported proximity to the nearest park and frequency of visits to parks/greenspaces). Non-human research, review articles or those with duplicate citations were excluded.

### Data extraction and quality assessment

The results of the searching process were exported to Endnote X9 (Clarivate Analytics, USA) software. All records’ titles, and abstracts were assessed for relevancy at first, and the irrelevant articles were omitted; then the full texts of the remaining articles were evaluated. The consolidated standards of the Newcastle-Ottawa quality assessment scale (NOS) were used to assess the quality of study design, sampling strategy, and measurement quality [45].

Data were extracted as follows: first author’s name, publication year, place of study, type of study, population, total sample size, mean age, type of measure, and other complementary information. Two independent research experts followed all searches, refinements, quality assessments, and data extraction processes. Any disagreements were resolved through consensus with the third investigator (Kappa statistic for agreement for quality assessment; 0.92).

### Statistical analysis

Meta-analysis was performed to estimate the combined effect sizes of (1) proximity, (2) access/availability, and (3) greenspace NDVI on CMRFs. A combined effect size was estimated in cases of more than two reports of the same exposure, outcome, and measure. To combine the association of the aforementioned variables with CMRFs as a dichotomous variable, first the ORs were standardized, and only standardized ORs and their 95% confidence interval (CI) were used as the effect size in the meta-analysis. We standardized the ORs to a 0.1 increase in NDVI, and for proximity to a 1000 m (1 Km) distance to green space by using the formulas described previously by Zhao et al. [46] Heterogeneity was assessed by the I^2^ and Cochran’s Q tests; if heterogeneity was statistically significant (Cochran’s Q *P*-value < 0.1), a random-effect model was adopted; otherwise, a fixed-effects was used for analysis. Publication bias was assessed using Egger’s test; if publication bias was significant, sensitivity analysis (trim fill analysis) (16) was performed. A two-tailed *p*-value below 0.05 was considered statistically significant. Stata version 17 (StataCorp. 2021. Stata Statistical Software: Release 17. College Station, TX: StataCorp) was used to analyze the data.

### Ethical considerations

The present study was approved by the ethical committee of the Alborz University of Medical Science. All included studies are cited in all reports and complementary extracted publications. We contacted the corresponding author whenever we needed more information about a certain study.

## Results

### Study selection process and study characteristics

The flowchart summarizes the study selection process for review (Fig. 1). The initial search of the database yielded 3839 hits (PubMed: 2543, Scopus: 936, ISI: 360). Duplicate studies through all databases were removed (*n* = 2926). After excluding ineligible articles through screening titles and abstracts, a total of 913 articles underwent a full-text evaluation. Finally, 118 articles met the inclusion criteria and were included in our review (i.e.,67 studies evaluated the association between greenspace and BMI, 30 evaluated the relationship between greenspace and HTN, 16 investigated the association between greenspace and BG, and 8 reviewed the association between greenspace and lipid profile or dyslipidemia). A summary description of included studies is presented in Table 1. All articles were published between 2005 and 2023 (50 in the last 5 years) The majority were published in North America (*n* = 43) [17, 26, 47–87], followed by Europe(*n* = 41) [12, 16, 21, 25, 88–124], Asia (*n* = 18) [13–15, 23, 27, 32, 35, 125–135], Oceania(*n* = 9) [136–144], South America(*n* = 6) [145–150] and Africa (*n* = 1) [132]. These studies had sample sizes ranging from 73 to 97,574,613 individuals (total number of participants = 112,719,774). A majority of included studies used cross-sectional design (*n* = 79, 66.9%), followed by prospective or retrospective cohort designs (*n* = 29, 24.5%), and some without mentioning the study type (*n* = 9, 8%). Almost 1/3 of the studies in this review (*n* = 37, 31.9%) included children as their target group while 45.7% of studies (*n* = 54) focused on adults. It should be noted that Browning’s study [54], which has the largest population (*n* = 97,574,613), used data from the US Centers for Disease Control and Prevention. Out of 118 reviewed studies, it has been shown that 49 studies only studied urban areas and 5 studies only include rural areas. 27 studies include both urban and rural areas. 4 studies have focused on urban and suburban areas. Only one study, in addition to the urban and rural areas, had also examined the suburbs. 32 studies have not mentioned the scope of the study as urban and rural and the scope of one study was unknown (Table 1).

### Characterizing exposure to greenspace

The majority of studies included in this review considered the proximity(distance to nearest greenspace) of the parks and greenspaces (*n* = 39, 37.8%) or (accessibility/availability) to greenspaces (*n* = 39, 37.8%); eighteen studies evaluated greenness and its density using normalized difference vegetation index (NDVI) [23, 27, 52, 54, 75, 83, 84, 112–116, 130–133, 143, 151]; The most commonly used methods to measure the greenspace characteristics were Geographic Information System (GIS), and Global Positioning System (GPS) (*n* = 53, 51.4%).

### The association between greenspace and weight status

#### Accessibility or greenness of greenspace and weight status

Overall, 31 articles (46.2%) [48–50, 52–54, 58, 61–64, 66–69, 75, 77, 78, 82, 92, 93, 96, 97, 102, 104, 108, 127, 138, 140, 147, 150] assessed accessibility to greenspace as a measurement, and these studies looked at the relationship between accessibility to greenspace and BMI in distinct target populations. Eight of the 31 studies (25.8%) [52, 54, 63, 67, 77, 97, 104, 140] found a negative association between BMI and access to greenspace Accessibility. Other studies in this review found no significant association between access to greenspace and BMI, as shown in the table (Supplementary Table 1). Some reviewed studies revealed varying effect sizes for subgroups such as men and women, low-income versus high-income populations, and various BMI sub-groups.

#### Proximity to greenspace and weight status

Proximity to nearby greenspaces was reported in 34 (50.7%) of the reviewed studies on BMI. Eight (23.5%) of the studies looked at the proximity of greenspace within a one-kilometer radius of the participant’s homes, and these studies found a negative correlation between BMI and proximity to greenspace [51, 59, 65, 70, 71, 76, 128, 141].

### The association between greenspace and HTN

#### Accessibility or greenness of greenspace and HTN

Eleven articles evaluated accessibility to greenspace as a parameter [12–14, 16, 18, 25, 26, 47, 89, 90, 139], and these studies assessed the relationship between accessibility to greenspace and HTN in different target groups. Almost all of these studies revealed a negative relationship between HTN and access to greenspace (Supplementary Table 2).

#### Proximity to greenspace and HTN

Three (*n* = 3) [15, 17, 137] of the five studies [15, 17, 88, 129, 137] that examined the relationship between proximity to greenspaces and HTN or cardiovascular health status found a positive relationship between lower proximity and higher blood pressure.

### The association between greenspace and blood glucose

Among all included studies (*n* = 16) that investigated the association between greenspace and BG levels or diabetes mellitus (DM), thirteen studies (81.2%) used the diagnosis criteria for DM by fasting plasma glucose(FPG) or HbA1c level, and the remaining studies reported incidence or mortality attributed to DM (Supplementary Table 3).

#### Proximity, accessibility to greenspace or greenness and blood glucose

Eleven studies that assessed the association of BG or diabetes with greenspace (i.e., proximity, greenness or accessibility) showed a negative association between greenspace and blood glucose level or diabetes status, and three studies found a positive association between these variables [31, 139, 152].

### The association between greenspace and lipids

Four studies (50.0%) among eight included studies assessed the relationship between public greenspaces and dyslipidemia, while the remaining studies investigated the association of greenspaces with a mean level of lipid profile (Supplementary Table 4).

#### Accessibility or greenness of greenspace and blood lipids

Five studies that assessed the association of dyslipidemia with greenspace (i.e., proximity, greenness or accessibility) showed a negative association between greenspace and dyslipidemia [23, 26, 27, 132, 151], and two studies found a positive association between these variables [116, 139].

### Quantitative synthesis

The combined standardized ORs of the association between greenspace and CMRFs are shown in Table 2. Our meta-analysis indicated that access to green space was associated with decreased the odds of DM by 21% (OR:0.79 95% CI (0.67,0.90)), HTN by 19% (OR:0.81 95%CI (0.61,1.00)) and obesity by 17% (OR:0.83 95%CI (0.77,0.90)). Moreover, 0.1-unit change in the mean NDVI and 1Km difference in NDVI decreased the odds of HTN by 9 and 21%, (OR: 0.91 95%CI (0.88,0.94)) and (OR:0.79 95%CI (0.61,0.98)) respectively. Proximity of 1Km and 15-minute walk to green space decreased the odds of obesity by 3% (OR: 0.97 95% CI (0.94,0.99)) and 51% (OR: 0.49 95%CI (0.02,0.99)) respectively.

**Table 2:** Meta-analysis of the association between greenspace exposure and CMRFs

reported CMRFs	measure (base of measure)	Number Of Studies	Combined standardized ORs ( 95%CI)	Heterogeneity Assessment		
				I Squared%	Model	*P*-Value
DM	access (availability)	3	0.79 (0.67,0.90)*	4.49	fixed	0.35
HTN	access (availability)	3	0.81 (0.61,0.99)*	83.1	random	0.001>
HTN	access (one kilometer)	5	0.99 (0.96,1.02)	0	fixed	0.62
HTN	NDVI (one kilometer)	5	0.79 (0.61,0.98)*	93.75	random	0.001>
HTN	NDVI (mean NDVI)	5	0.91 (0.88,0.94)*	71.34	random	0.01
Obesity	access (availability)	16	0.83 (0.77,0.90)*	66.92	random	0.001>
Obesity	access (one kilometer)	3	0.98 (0.82,1.14)	90.46	random	0.001>
Obesity	proximity (one kilometer)	8	0.97 (0.94,0.99)*	44.39	random	0.07
Obesity	proximity (15 min walk to the park)	3	0.49 (0.02,0.99)*	90.51	random	0.001>

CMRFs: cardiometabolic risk factors, DM: diabetes mellitus, HTN: hypertension, NDVI: normalized Difference Vegetation Index, OR: odds ratio, CI: confidence interval

### Publication bias

Publication bias was assessed across studies assessing greenspace and CMRFs. However, no publication bias was seen among the studies (*P* > 0.05).

## Discussion

**Fig. 2:**
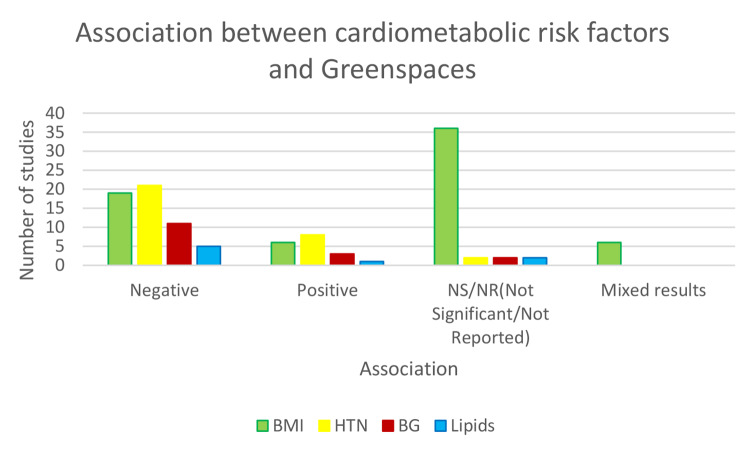
Association between greenspace exposure and cardiometabolic risk factors in reviewed articles. NS/NR = not significant/not reported

**Fig. 3:**
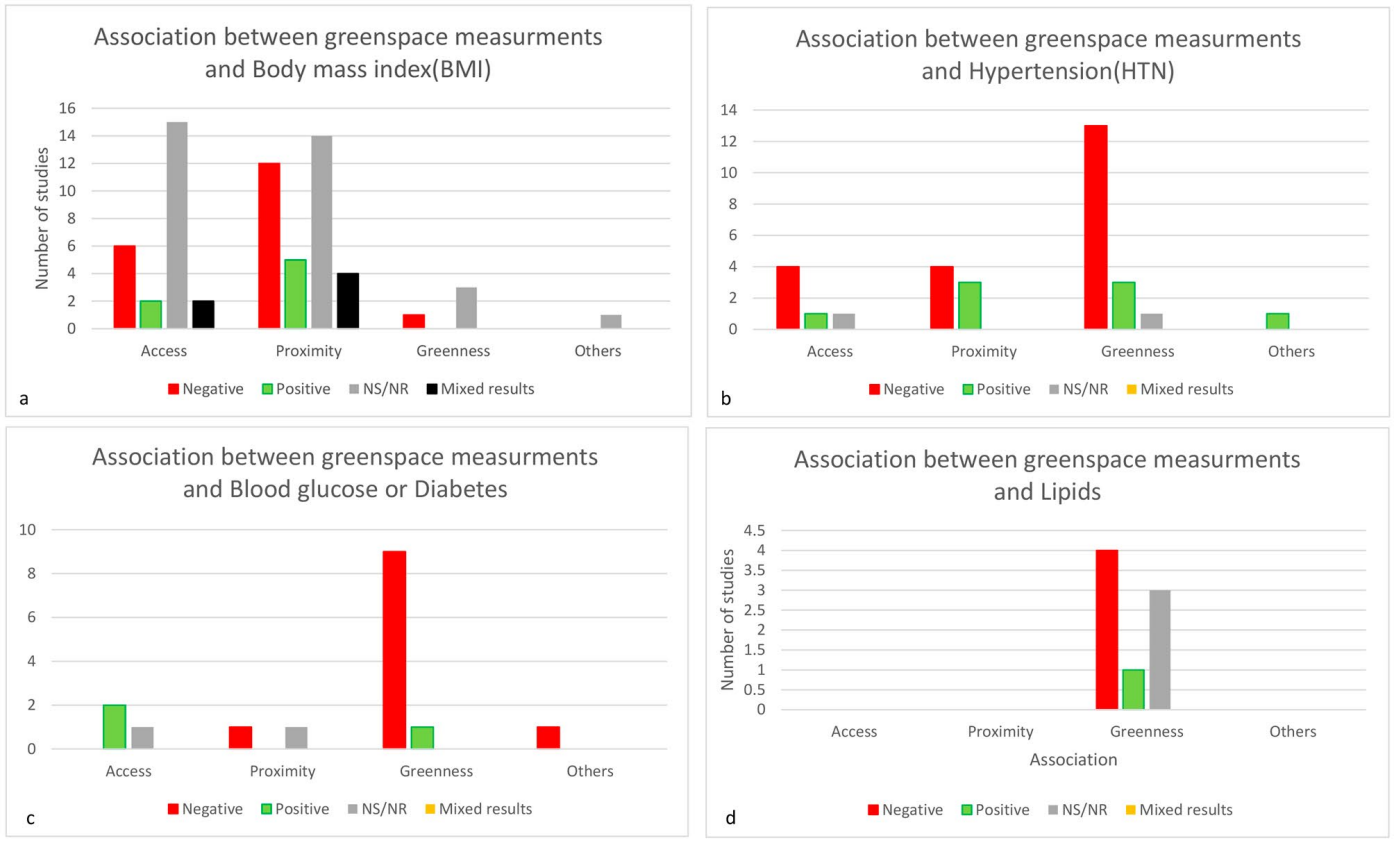
**a**: Association between greenspace and Body mass index (BMI), **b**: Association between greenspace and Hypertension (HTN),**c**: Association between greenspace and blood glucose(BG),**d**: Association between greenspace and lipid profiles in reviewed articles

In this paper to the best of our knowledge, we reviewed for the first time studies based on access to greenspaces and public open spaces (POS) and their associations to cardiometabolic risk factors such as obesity, HTN, dyslipidemia and diabetes. Considering we are experiencing an epidemic of cardiometabolic risk factors, primarily in metropolitan regions with fewer outside activities, this is a novel study that examines the relationship between access to POS and greenspaces and CMRFs. This is indeed a new area of research, so this report pointed out exclusively 118 papers, nearly half (*n* = 57, 55.3%) of which were published in the last six years (2016_2022). All the other studies, except for eleven [49, 52, 53, 58, 60, 80–82, 97, 104, 108] were carried out over the last decade. This matter demonstrates that, given the worldwide obesity epidemic and the hot topic of the related cause of this epidemic, our review topic depicted one associated probable environmental etiology of obesity. In this review, we also looked at HTN as a leading cause of cardiovascular disease and major mortality, as well as the impact of greenspace and POS on other important CMRFs like BG, diabetes, lipid profile levels and dyslipidemia. This systematic review comprised 118 studies from 13 countries, with developed countries accounting for 91.3% (74 papers). More than half of the articles reviewed (51.1%) identify and analyze correlations between greenspace and obesity or being overweight. The current review is among the first systematic reviews to look at the effect of greenspace proximity or accessibility on individuals’ BMI, HTN, DM, and dyslipidemia in recent years (Fig. 2). Around half of the papers (36 out of 67) observed no significant relationship or some weak or mixed corroboration of an association between greenspace and BMI, 19 out of 67 confirmed a negative relation with BMI, and 6 papers revealed a positive association with BMI (Fig. 3a).

Moreover, eleven studies found a negative relationship between HTN and greenspace; five studies found a positive relationship (Fig. 3b). Furthermore, eleven studies found a negative relationship between BG or DM and greenspace, three studies found a positive relationship, and the remaining studies that assessed BG or DM found no significant relationship between these two indicators (Fig. 3c). These papers with uncertain results were removed from the review during the study selection process, or if they met the inclusion criteria, they did not provide strong evidence of the relationship between greenspace and BMI or HTN. By the results of our meta-analysis, green space access reduced the odds of diabetes mellitus by 21%, hypertension by 19%, and obesity by 17%.

### Health and food literacy and association with greenspace accessibility

The findings revealed that parents with a low Health literacy were nearly twice as likely to report that their newborn spent time in front of the television, and three times more likely to report excessive daily “tummy time [153].”

The study found that adolescents who were members of sports groups had a greater health literacy than nonmembers, independent of age or gender [153].

The majority of the studies reviewed found a positive connection between health literacy and physical activity, which can be explained by the fact that people with higher levels of health literacy have the skills and capabilities to engage in a variety of personal health-enhancing behavior, such as regular physical activity [153–155]. This could also have explained the link between health literacy and greenspace utilization for physical activities and outdoor exercises.

Studies indicate that children’s and parents’ misperceptions regarding their children’s weight are caused by a lack of parent-child communication about health issues, unhealthy weight self-management behaviors, and a delayed approach to weight problems and late interventions [156, 157].

Efforts should be directed toward developing school-based programs that assess children’s weight and accurately communicate their nutritional status to both children and parents, as well as actions to improve food literacy and physical activity literacy, making better use of available green spaces and open public spaces (POS), to mitigate the youth obesity epidemic and lower cardiometabolic risk factors.

### The resemblance with systematic reports and viewpoints

#### Greenspaces and BMI

Seven prior pieces of the literature identified a connection between greenspace and overweight/obesity [38, 39, 41, 158–160] (Fig. 3a). Dunton et al. published a systematic review of the relationship between the physical environment and childhood obesity in 2009 [39]. In two cross-sectional studies of 245,000 Australian adults over 45 years old, an increasing proportion of land in the neighborhood covered by greenspace was associated with a lower risk of overweight, obesity, and diabetes [10]. Only 13 studies were found to evaluate the relationship between greenspaces and individuals’ weight status in a review study published in England, the majority of which were performed in the United States [11].

This study also determines the built and biophysical environmental variables that are linked to childhood obesity and physical activity levels. Using fifteen eligible studies on this topic, we found that childhood obesity and physical environmental variables differed depending on gender, age, socioeconomic status, population density, whether the reports were made by children themselves or their parents. Obesity outcomes in adolescents were associated with access to equipment and facilities, neighborhood patterns, and urban sprawl, according to this review. This study found no association between the number/distance to parks, as well as the presence of parks, and BMI [39]. Lachowycz et al. [9] reviewed 16 studies on greenspace and obesity. The majority of the studies reviewed in this article yielded inconclusive results regarding the relationship between greenspace access and obesity-related health indicators. Several studies have also revealed some variables that may influence this relationship, such as age, socioeconomic status, and greenspace measurement. This review looked at studies that primarily used BMI as a weight status indicator [9].

D-Mackenbach et al. published a review on obesogenic environments in 2014. Five databases were systematically searched for studies published between 1995 and 2013. This systematic review discovered two components: urban sprawl and land use mix, which are Inextricably linked to weight status [160]. Greenness and its health benefits were reviewed by James et al. in 2015 [159]. This review revealed relatively solid evidence for a positive connection between greenness and physical activity and a less consistent negative relation between greenness and body weight [159]. Maike Schulz et al. performed a systematic review of the build-up environment and health in Germany in 2018. This study examined 25 papers relating to the use of sport-related physical activities; however, it was not related to body composition [158].

Sabine Jean-Louis et al. published a systematic review of the relationship between greenspace access and obesity in 2018. This review has illustrated that 80% of the studies assessed; have shown a positive correlation between these two [41]. Ya-Na Luo et al. published a systematic review and meta-analysis on greenspace and obesity in 2020. This review looked at a total of 57 studies on the subject. More than half of these studies identified a connection between greenspace and lower levels of overweight/obesity [38]. In comparison, the study results of our systematic review point in the same direction as the findings of previous reviews. The majority of previous reviews assess levels of physical activity, but this is not the main character in our review. Other reviews have evaluated the anthropometric parameters of weight status, but the main character in our study was BMI, and other variables were not reviewed in our evaluation. As a result, we reviewed 45 studies on the relationship between greenspace and BMI. The evaluation revealed that in these studies, greenspace is defined as access to greenspace and proximity to greenery. The BMI was our primary parameter for assessing weight status in adults, adolescents, and children. Our review primarily uses OR reports to assess the relationship between BMI and greenspace. As a result, we believe that our systematic review evaluates previous studies on this topic, as well as our tables and documentation in our findings; 19 studies found a significant negative relationship between BMI and greenspace measurements [51, 52, 62, 63, 65, 67, 70–72, 74, 76, 77, 106, 136, 145, 149, 161, 162]. These findings indicated that increased access to greenspace could lead to lower BMI, but inconsistency in the age groups of the studies reviewed and different covariates make widespread generalization difficult. The same probably applies to previous research. Previous studies on greenspace and its effect on BMI produced contradictory results. Our findings also revealed the same inconsistency, which was most likely caused by measurement heterogeneity.

#### Greenspaces and HTN

This review compiled indications of the associations between greenspace and HTN (or blood pressure). We evaluated 30 articles on the topic of HTN and greenspace in this review. A systematic review of greenspace and health in Mainland China investigated the association between health status, mental health, weight status, cardiometabolic outcomes, and greenspace. Seven of the 14 studies in this review looked at HTN as a cardiometabolic outcome, and all of the cross-sectional studies found a negative relationship between HTN and greenspace measurements [42] (Fig. 3b). Almost all of the articles reviewed were published within the last four years (2016–2020). Furthermore, more than half of the studies (*n* = 9, 60%) were conducted in Europe or The United States, while one-third were conducted in Asia or Oceania. (*n* = 5, 33.3%) One study included in this review assessed pregnant women and their children and followed them up to the age of eight years [17]. According to Jimenez et al. study Living one mile farther away from a greenspace at birth was associated with 5.6 mmHg higher adult SBP (95%CI: 0.7, 10.5), and 3.5 mmHg higher DBP in adjusted models (95%CI: 0.3, 6.8). One more greenspace in the neighborhood at birth was also associated with lower DBP in adulthood (− 0.2 mmHg, 95%CI: −0.4, − 0.02) [17]. In addition, two studies looked at the relationship between greenspace and HTN in children [15, 110]. The remaining articles focused on adult populations as target groups. Abbasi et al. discovered lower SBP and DBP in children who lived near greenspaces (− 0.08mmHg and − 0.09 mmHg, respectively), but these findings were not statistically significant in the ORs reported for isolated elevated SBP, DBP, and HTN. This could imply that more research is needed to determine whether the results are supportive or not [15]. In the study by Markevych et al., they also evaluated children aged 10 years old and discovered that lower residential greenness was positively associated with higher blood pressure in 10-year-old children living in urban areas. This finding requires further investigation to confirm the theory of greenspace’s effect on children’s blood pressure and to assist policymakers in providing more public open spaces and greenspace for children in urban areas to reduce the risk of HTN in their adulthood [110]. A study by Bijnens et al. focused on twins aged 18 to 25 years old to see if there was an association between HTN and greenness in this population. They discovered that a 3.59 mmHg (95% CI:-0.6 to -1.23; *p* = 0.005) decrease in adult night systolic blood pressure was associated with an interquartile increase in residential greenness exposure (1000 m radius). Night-time blood pressure was inversely related to residential greenness in adulthood and residential greenness in childhood in twins who lived at a different address than their birth address at the time of the measurement (*n* = 181, 65.1%) [12]. This could clarify the effect of greenness in the living area regardless of age. Since the majority of the reviewed articles assessed accessibility to greenspace, these articles primarily discovered a negative association between HTN and accessibility to greenspace, as detailed in Supplementary Table 2. Studies that evaluated greenspace based on their proximity [15, 16, 88, 137] found a positive relationship between proximity to greenspaces and higher SBP, DBP, or HTN [15, 16, 88, 137]. This review included a study evaluating the effect of HTN and greenspace in early pregnancy. This study found a positive association between proximity to greenspaces and HTN in pregnant women [88]. As previous studies showed that women in their first trimester of pregnancy are an appropriate group for the study of hypertensive disorders because, while changes in pregnancy cause increased stress on the cardiovascular system, such effects primarily occur from the second trimester of pregnancy [163]. As a result, blood pressure during the first trimester of pregnancy is primarily caused by external factors [88, 163].

#### Greenspace and BG

This review compiled indications of the associations between greenspace and blood glucose levels or DM. We evaluated 16 articles on the topic of BG or DM and greenspace in this review. Almost all of the studies applied greenness as a greenspace measurement, and 11 of them found a negative association between BG levels, DM diagnosis, or the prevalence of T2DM, while three found a positive association [31, 139, 152] (Fig. 3c).

Ruijia Li et al. showed that an increase in the NDVI within a 500 m buffer radius is associated with a 13.4% decrease in FBG with an odds ratio (OR) of 0.866 and 14.2% (OR: 0.858) decreased risk of T2DM [130].

According to Ngam et al., greenspaces with sports facilities have a significant relationship to cerebrovascular diseases; the most distant population had an 11% higher prevalence rate ratio (PRR) of cardiovascular diseases (CVD) than the nearest, as well as a 9% higher diabetes risk (PRR) than the nearest [152].

Liao et al. found that living in areas with more greenspace was associated with lower maternal glucose values and a lower risk of incident maternal impaired glucose tolerance (IGT) and gestational diabetes mellitus (GDM) [131].

Dadvand et al. revealed an inverse correlation between time spent in greenspaces, specifically natural greenspaces, and FBG levels; and an increase in total time spent in greenspaces of 1.83 h was associated with a 0.5 mg/dl decrease in FBG levels in children aged 7–18 years [35].

#### Greenspace and lipids

This review collected information on the associations between greenspace and lipid profile levels, also known as dyslipidemia. In this review, we focused on 8 articles about lipid profile levels or dyslipidemia and greenspace. Greenness was used as a greenspace measurement in all of the studies, and four of them found a negative association between lipid profile levels and dyslipidemia, while one found a positive association [116] (Fig. 3d).

According to Iyer et al., a 0.11 unit increase in NDVI was associated with lower BMI and diabetes, but there was no association between NDVI and hypertension or cholesterol [132]. Residential greenness was associated with an increased risk of dyslipidemia in Chinese rural-dwelling adults, particularly among males, according to a study by Jiang et al. [116].

### Probable mechanisms

Despite widespread agreement that physical environments and access to public open spaces such as vegetation play an important role in people’s weight status, a large body of research has failed to identify direct associations between greenspace and obesity. Here are some hypotheses that could explain this association. Greenspace can boost physical activity through both walking and cycling routes, as well as places to exercise and play [164]. Greenery is strongly correlated with more outdoor playing in children [165]. In addition, the risk of ambient air Pollution and noise may be reduced by vegetation. There is evidence of the possibility of obesity due to air pollution [25]. According to recent studies, the availability to greenspace and exposure to mixed bacteria may help to prevent obesity as an inflammatory disease by balancing the immune system to prevent inflammatory processes like obesity [24, 166]. Individuals’ stress levels may be reduced, and their social cohesion may be increased if they have easy access to greenspace [25]. This finding lends credence to the Glonti et al. study’s finding that people with higher levels of social cohesion have a lower risk of obesity [167].

Although the mechanisms by which greenery improves health and HTN remain unknown, several biopsychosocial pathways have been proposed [25]. Stress reduction and recovery, increased physical activity, social cohesion endorsement, and reduced exposure to air pollution and noise have all been suggested as possible mechanisms in the green-health pathway, all of which could be essential in evaluating the risk of HTN in urban populations [12, 26, 168–171]. According to facts, adiposity, a well-documented risk factor for HTN, appears to be reduced in green environments. The findings support this hypothesis, which shows that BMI mediated a large portion of the association between greenness and blood pressure [172]. Greenness has also been associated with lower noise and heat exposure, enhanced social cohesion, greater and more diverse microbial exposure, and lesser psychological and physiological stress [24, 25].

Using greenspaces in a neighborhood can be beneficial for physical activity. This means that it should be easily accessible and promoted for active use [115]. Despite these findings, the relationship between greenspace exposure and incident diabetes is not fully understood. Besides physical activity, other explanations may exist, for example, the benefits of exposure to nature for immunological regulation [24].

While the biological mechanisms underlying greenness’s beneficial effect on blood lipids are unclear, previous research has suggested several biopsychosocial pathways, including reduced levels of air pollution [151, 173, 174] and increased physical activity [23, 175, 176], which could reduce lipid peroxidation products and oxidative stress markers and further improve lipid profiles [23].

### Limitations and strengths

Various limitations should be implied for proper interpretation of our systematic review. First, data in included studies in terms of exposure and outcome definition and measurement was severely heterogeneous, which could bias the final interpretation. Second, there was not enough data from developing countries, and due to the increasing growth of obesity, diabetes, HTN and dyslipidemia in these nations and also different socioeconomic and geographic information, these results cannot be generalized to these nations. Third, statistical modeling methods differed significantly, with several cofounding factors evaluated by different studies. As a result, some studies may have over-adjusted or under-adjusted for confounding factors, resulting in biased effect estimates. Fourth, only anthropometric measures representing weight status in studies were used in this systematic review, including BMI, which may bias the results. Other anthropometric characteristics may more accurately represent weight status. Fifth, the majority of the studies reviewed in this article were cross-sectional, which may impact the possible association in case follow-up, whether retrospectively or prospectively. Despite the limitations mentioned, this article has systematically studied the effects of greenspace on the CMRFs of the people studied in various articles. The general population provided a sufficient sample size, and despite the lack of sufficient data from developing countries, the studies examined were successful. Therefore, our results may be helpful for experts in the field of greenspace overweight/obesity, and policymakers in the field of developing a strategic plan to mitigate the burden of obesity.

Almost all of the studies reviewed in this article related to HTN were conducted within the last four years, but further research in different age groups is needed to confirm the findings of the relationship between HTN and greenspace. Only three studies, two with a target population of children [15, 110] and one with a target population of pregnant women [88], were chosen for this article to evaluate the association between blood pressure and greenspace. More research is needed to generalize these studies’ findings. In addition, four studies [47, 89, 90, 137] focused on cardiovascular events and health status in general as an outcome of the research. The results were not specific enough for this review article to evaluate greenspace’s effect on blood pressure.

Almost all of the studies reviewed in this article related to DM or dyslipidemia were conducted within the last four years, but further research in different age groups is needed to confirm the findings of the relationship between DM or dyslipidemia and greenspace. The obscured mechanism linking greenspaces and diabetes or dyslipidemia by increasing physical activity could be due to measurement error in exposure and outcome, residual confounding between greenspace and diabetes risk, and the fact that we had an overall measure of physical activity rather than just that done in greenspace [23].

Almost all of the studies reviewed in this paper were conducted in Urban regions and metropolitan areas so there are some limitations for comparing rural and urban areas for association of CMRFs and access to greenspaces. And also since there is more access to POS in urban areas with better socioeconomic conditions and people with better socioeconomic conditions in major metropolitan cities possibly access to better health literacy and leads to more physical activities [155]. So in this reviewed we have limitations for interpretations of the association of access to greenspaces and CMRFs in metropolitan cities.

### Recommendation for forthcoming reviews and studies

According to the limitations of our review, we recommend that future articles on this topic follow these steps to properly imply an association between weight status, HTN, diabetes, dyslipidemia and greenspace availability. First, future articles could assess the effect of greenspace on individuals’ physical activity and support the theory that better access to greenspace may lead to higher physical activity and, as a result, lower BMI and lower blood pressure. Future studies can also be conducted in different age groups, focusing on pregnant women and the effect of greenspace availability on their health status to determine whether access to greenspace is more effective in older or younger age groups. Finally, data from developing countries may alter the effect of greenspace on obesity and HTN and assist researchers in generalizing the relationship.

## Conclusions

According to the findings of this review, greater access to greenspace is associated with lower SBP/DBP or lower risk of HTN, as well as a lower chance of being overweight or obese with a lower BMI and lower BG levels and lipid profiles. Regardless, a firm conclusion cannot be drawn due to a large number of articles with no significant results, the extensive interplay between-study heterogeneity, and the small number of accessible studies.

### Electronic supplementary material

Below is the link to the electronic supplementary material.

Supplementary Material 1

## Data Availability

The data sets used and/or analyzed during the current study are available from the corresponding author upon reasonable request.

## Abbreviations

BG: Blood glucose
BMI: Body mass index
BP: Blood pressure
DBP: Diastolic blood pressure
HTN: Hypertension
SBP: Systolic blood pressure
