# Supplementary material for: Association of greenspaces exposure with cardiometabolic risk factors: a systematic review and meta-analysis
Source: BMC Cardiovasc Disord. 2024 Mar 20;24:170. doi: 10.1186/s12872-024-03830-1 (PMC10953288; doi:10.1186/s12872-024-03830-1)
Supplement: Supplementary file 1 — Supplementary Material 1 [file 12872_2024_3830_MOESM1_ESM.docx]

| Supplementary Table 1: Main finding of included studies about the association between greenspace exposure and BMI | | | | | | | | | | | |
| --- | --- | --- | --- | --- | --- | --- | --- | --- | --- | --- | --- |
| No | Authors (Year) | POS Characteristics | | | | Outcome | | | Finding | | |
|  |  | Type | | Definitions | Measurement | Type | Definitions | Measurement | Quantitative | Qualitative | Covariate adjustments |
| 1 | Zhang (2019)[83] | proximity | | Networked distance to the nearest park (Kilometer) | GIS  Google Maps | body mass index (BMI) | Continuous BMI | It was calculated as weight in kilograms divided by height in square  meter (kg/m2) | Adj. B coefficient (95% CI): −0.29 (−0.47, −0.10) | Negative | Age, gender, education,  country of birth, years of living in the United States, household income, health insurance coverage |
|  |  |  |  |  |  | Obesity | BMI ≥ 30 |  | OR adjusted (95% CI): 0.96 (0.90, 1.01) | NS |  |
|  |  |  |  |  |  | Morbid obesity | BMI ≥ 35 |  | OR (95% CI): 0.91 (0.85, 0.98) | Negative |  |
|  |  |  |  | Walking time to the nearest park | GIS  Google Maps | body mass index (BMI), | Continuous BMI | It was calculated  as weight in kilograms divided by  height in square  meter (kg/m2) | Adj. B coefficient (95% CI): −0.02 (−0.04, −0.01) | Negative |  |
|  |  |  |  |  |  | Obesity | BMI ≥ 30 |  | OR (95% CI): 1.0 (0.99, 1.00) | NS |  |
|  |  |  |  |  |  | Morbid obesity | BMI ≥ 35 |  | OR=0.1 (95% CI): (0.99, 1.00) | Negative |  |
| 2 | Vaccaro, (2019) [68] | Access/availability | | Parents reported playground and parks in Neighborhood | Interview | overweight/obese | z-BMI scores ≥ 85th percentile | Overweight or obese was constructed from the variable body mass index for age and sex percentiles (z-BMI) | OR (95% CI) [having access/ not having access]  : 0.98 (0.86-1.11) | NS | Sociodemographic, behavioral, and parental  factors |
| 3 | Astell-Burt (2014) [138] | Proximity | | Percentage of proximity to greenspace within a 1-km catchment area from each centroid | GIS | Overweight | BMI: 25–29.9 | Based on the Self-reported height and weight | Adj RR for women with over 80% proximity to green  space: 0.90 (95% CI: 0.83-0.97)  Not significant for men (RR not reported) | Negative for women  NS for men | Age, moderate-to-vigorous physical activity |
|  |  |  |  |  |  | Obese | BMI > 30 |  | Adj OR for women with over 80% proximity to green  space:0.83 (95% CI: 0.74-0.94)  Not significant for men (RR not reported) |  |  |
| 4 | U. Goldsby (2016) [55] | Proximity | | distance to Railroad Park from the child’s home  (Exposure group:  1.5 to 3  and 3 to 5 miles  Control Group:  > 5 miles) | GIS | Body Mass Index (BMI) z-score | Normal= 5th to 85th percentile | Measures of relative weight adjusted for child age and sex | RC (Near, within 1.5 miles) = -0.1895, SE =0.1656, OR Crude: 0.66 (95% CI: 0.28, 1.54), (Intermediate Between 1.5 and 3 miles) =0.1243, SE=0.0924; OR Crude: 1.13 (95% CI: 0.73, 1.74) (Intermediate, Between 3 and 5 miles) =0.0733;  OR Crude: 0.91 (95% CI: 0.64, 1.30) | NS | Age, gender, race, ethnicity, and payer  type |
|  |  |  |  |  |  |  | Overweight= 85th to 95th percentile |  |  |  |  |
|  |  |  |  |  |  |  | Obese≥ 95th percentile |  |  |  |  |
| 5 | Alexander (2013) [50] | Access/availability | | Places  and things are available to children in  neighborhood | Parents self-reported | Obesity | Obese ≥  85th percentile | Reported by the parents (telephone interview) | Adj PR: 0.77 (95% CI: 0.55-1.07) | NS | Age, race/ethnicity, maternal and paternal education, socioeconomic status, geographic location and living status. |
| 6 | Bai (2013) [51] | proximity | | The area  within a 10- to 15-min walk from home | 60 parks were selected as part of a related study to be  geographically  dispersed across Kansas City | BMI | Underweight (BMI<18.5) | Self-reported height and weight | BMI OR: 0.69, (95% CI: 0.54 – 0.88). | Negative | Age, gender, race/ethnicity, and past park use |
|  |  |  |  |  |  |  | Normal weight (18.5<BMI<25) |  |  |  |  |
|  |  |  |  |  |  |  | Overweight (25<BMI<30) |  |  |  |  |
|  |  |  |  |  |  |  | Obese (BMI>30) |  |  |  |  |
| 7 | Bell (2008) [52] | Access/availability | | Normalized difference  vegetation index NDVI within ≥1000 m of ≥1 park | Using the  NDVI | BMI z-scores | Continuous BMI z-score | Measured by clinic staff using scales and stadiometers | RC: B=–0.06 SD, (95% CI: –0.09–0.02)  OR 0.87 (95% CI:0.79, 0.97) | Negative | Racial/ethnic group; gender; age at baseline; and health insurance status; as a proxy for individual socioeconomic status(SES) |
| 8 | Bird (2016) [76] | Proximity | | Three closest parks within a 500 m walking network buffer around, If no parks  were found within 500 m, the walking buffer was increased to 1000 m | Can Map | BMI | Overweight and obese  ≥85th percentile | Weight, height and waist circumference were measured according to standard protocols | Compared to children living near parks that are small with no team  sports feature and no play area feature for children ages 6 years and  over, Children living near esthetically  pleasing parks with few team sports installations had less truncal fat, RC: −3.4 (95% CI: −6.4;−0.5]) | Negative | Household income, sex, age, and puberty |
|  |  |  |  |  |  |  | Obese  ≥95th  percentile |  |  |  |  |
| 9 | Burgoine (2015) [92] | Proximity | | Within our 800 m street network  home | GPS | BMI z-scores | continuous BMI z-score | Measured by trained research staff | RC: − 0.315 (95%: − 0.897-0.268) | NS | Parental education  level and sex of child |
| 10 | Dadvand (2014) [106] | Proximity | | Within  300 m separately from a park or forest | To measure surrounding greenness, we used the NDVI  Residential proximity to greenspaces as a surrogate for the access to greenspaces, Urban  Atlas map | BMI z-score | Overweight/obesity | Dividing weight (in kilograms) by height (in meters)  squared | Park RC: –0.07 (95% CI: –0.17, 0.03)  Forest RC: –0.06 (95% CI: –0.21, 0.10) | NS | Parental education, type of school, sport activity,  and having siblings |
| 11 | Davidson (2010) [77] | Access | | Existence of good playgrounds/parks | Interview | BMI | Normal | Dividing weight (in kilograms) by height (in meters)  squared | RC:  -0.261 (95% CI: -0.421--0.101) | Negative | Gender, household income, parental education and place of residence |
|  |  |  |  |  |  |  | Overweight |  |  |  |  |
|  |  |  |  |  |  |  | Obese |  |  |  |  |
| 12 | Gose (2013) [99] | Proximity | | Distance to the nearest park  within an 800 m | GIS | Children’s BMI-SDS | Overweight inclusive obesity ≥ 25 kg/m² | Calculated (kg/m 2) and the 90th age and  sex-specific German BMI percentile was used to define overweight including obesity | Social factors rather than neighborhood  environment (especially social environment) had an impact on children’s BMI-SDS over 4 years | NS | _ |
| 13 | De Assis (2018) [147] | Proximity | | Park Does not have  Up to 10 min  11 to 20 min  More than 20 min  Distance | Children and adolescents  aged 6 to 15 years, Neighborhood Environment Walkability Scale | BMI  overweight | Overweight | Trained researcher using a calibrated digital scale (weight in kg)  and a portable stadiometer (height in m) | up to 10 minutes to park, OR: 0.21 (95%CI: 0.06–0.81) | Negative | Gender and age of the child or adolescent, schooling of the parent or guardian, household monthly income, and Health Vulnerability Index |
|  |  |  |  |  |  |  |  |  | 11 to 20 min to park, OR: 1.54 (95% CI: 0.53–4.45) | NS |  |
|  |  |  |  |  |  |  |  |  | More than 20 min to park, OR: 0.15 (95%CI: 0.01–1.50) | NS |  |
| 14 | Hobbs (2018) [93] | Access | | 2 km radial buﬀer  (Low access & moderate access) | ArcGIS | BMI | obesity  BMI ≥ 30 | Using self-reported height and weight; | Moderate availability, OR: 1.18 (95% CI: 1.05-1.32) | Positive | Age, gender, ethnicity  (White-British and other), deprivation score (Index of Multiple  Deprivation) and rural or urban classification |
| 15 | Klompmaker (2018) [100] | Proximity | | Park distance:  ≤100 m  101–200 m  201–300 m  301–400 m  401–500 m  501–1000 m  > 1000 m (reference group) | Network distance to park entrance  NDVI and land-use databases  ArcGIS | BMI | Normal ≤ 24.9 kg/m^2^ | Self-reported height and weight were used to calculate BMI | ≤100 m  OR: 1.00 (95 % CI: 0.96, 1.05) | NS | Age, gender, marital status, country of origin, education, work, income,  smoking, alcohol, indoor physical activity, neighborhood SES and degree of urbanization |
|  |  |  |  |  |  |  | Overweight≥ 25.0 kg/m^2^ |  | 101-200 m  OR: 1.02 (95% CI: 0.98, 1.06) | NS |  |
|  |  |  |  |  |  |  |  |  | 201-300  OR: 1.03 (95% CI: 0.99-1.07) | NS |  |
|  |  |  |  |  |  |  |  |  | 301-400  OR: 0.99 (95% CI: 0.95-1.03) | NS |  |
|  |  |  |  |  |  |  |  |  | 401-500  OR: 1.01 (95% CI: 0.97-1.05) | NS |  |
|  |  |  |  |  |  |  |  |  | 501-1000  OR: 0.95 (95% CI: 0.92-0.98) | Negative |  |
| 16 | Lovasi (2011) [61] | Access | | Based on the proportion of land area covered, using 2003–2005 data  provided by the New York City Department of Parks and Recreation  0.5 km buffer around | GIS | BMI z-scores | Normal (BMI below  85th percentile) | The child was weighed on a portable Seca electronic scale  and height was measured using a portable Seca stadiometer base on CDC | _ | NS | Age, sex, and race/ethnicity), mother (age, born outside of the USA, use of Spanish, employed/student status),  household (number of rooms) |
|  |  |  |  |  |  |  | Overweight (BMI 85th  to 94th percentile) |  | _ |  |  |
|  |  |  |  |  |  |  | Obese (BMI at or above  95th percentile) |  | RC: − 0.04 (95% CI: − 0.19 to 0.11) |  |  |
| 17 | Manandhar (2019) [127] | Access/availability | | Accessibility to a park  (No, Easy) | GPS | Children BMI |  | Interviewed | OR: 0.58 (95 CI: 0.03-8.86) | NS | NR |
|  |  |  |  | Use of a greenspace (often, Seldom) |  |  |  |  | OR: 3.11 (95% CI: 0.49-19.63) | NS |  |
|  |  |  |  | Distance to greenspace  (< 1 km, >1 km) |  |  |  |  | OR: 27.46 (95% CI: 6.10-123.54) | Positive |  |
| 18 | Mathis (2017) [62] | Access/availability | | Availability of a park in the  neighborhood (yes/no) | Geographic Products Branch U.S. Census Bureau | Adult BMI | Normal  weight (<18.5–24.9) | Self-reported weight and height | Reference | _ | NR |
|  |  |  |  |  |  |  | Overweight (25–29.9), |  | OR: 1.18 (95% CI: 0.43, 3.21) | NS |  |
|  |  |  |  |  |  |  | Obese (30+) |  | OR: 0.37 (95% CI: 0.14, 0.98) | Negative |  |
| 19 | Mena (2014) [148] | Proximity | | Refers to the straight-line distance from the participant’ s home to the nearest public place for physical activity (i.e., parks) | GIS | BMI | Overweight  ≥ 25 and < 30 kg/m2 | = weight/height  2 | SC:  BMI was signiﬁcantly and positively related to the distance to  parks (ρ = 0.079, p < 0.05),  Waist circumference was similar and positively  related to distance to parks (ρ = 0.097, p < 0.05) | Positive | Age and gender |
|  |  |  |  |  |  |  | Obese  ≥ 30 kg/m^2^ |  |  |  |  |
|  |  |  |  |  |  | Abdominal obesity with waist circumference | > 102 cm in men |  |  |  |  |
|  |  |  |  |  |  |  | > 88 cm in women |  |  |  |  |
| 20 | Mendes (2013) [149] | Access/availability | | Parks (Yes /No) | GIS | BMI | Normal  BMI < 25 kg/m^2^ | Interviewed | PR:  0.99 (95% CI:0.72, 1.37) | NS | NR |
|  |  |  |  |  |  |  | Overweight  BMI ≥ 25 kg/m^2^ |  |  |  |  |
| 21 | Hughey (2017) [165] | Access/availability | | Availability was calculated as the number of each  facility within or intersecting each youth's Census block group | GIS | Children BMI |  | Height, weight, date of birth, and date of testing were used to calculate  body mass index (BMI) percentiles | RC: 1.5 (0.08) for men  RC: -2.2 (0.9) for women | NS for men  Negative for women | NR |
| 22 | Grit Müller,(2017)[101] | Proximity | | Distance of 800 m to the closest park or forest | Network  analysis based on the street network  for the minimum distance from the participants’ residential addresses to the closest park or forest | BMI | obesity (BMI ≥ 30 kg/m 2) | Weight and height were measured during physical examination | MRC:  0.33 (95% CI: -0.21 to 0.88) | NS | Age, sex, migration background, living with a partner, education, income, neighborhood unemployment rate |
| 23 | Nicolle-Mir (2018) [108] | proximity | | Distance to a city park ≤300 m  >300 m | NDVI measurement cut-off point by median | Children BMI | BMI ≥ 18 kg/m^2^ | the child’s weight (in kg) divided by height (in m^2^) | OR=  1.72, (95% CI)  1.15–2.60 | NS | - |
|  |  |  |  |  |  |  | BMI < 18 kg/m^2^ |  |  |  |  |
| 24 | Nies (2015) [79] | Proximity | | Proximity, measured in meters, to green  space,  maximum search distance was consistently set to 10,000 meters. | GIS | BMI | Overweight >25 kg/  m^2^  Obesity >30 kg/  m^2^ | Height and weight were recorded  independently in the EHR, | Mixed model analysis: 00395, standard error: 00352  P-value=0.2628 | NS | Sex and age |
| 25 | Picavet (2016) [91] | Access/availability | | Percentage of green within  125 m radius. | National Land Cover Classiﬁcation Database | BMI | Overweight 25–30 kg/m^2^ | Measurement by trained  personnel during a visit to the municipal health service. | OR: 1.01 (95% CI: 0.99; 1.03) | NS | Age, sex and socioeconomic status |
|  |  |  |  |  |  |  | Obesity N 30 kg/m^2^ |  | OR: 1.04 (95% CI: 1.01; 1.07) | Positive |  |
|  |  |  |  | Percentage of green within 1 km radius |  |  | Normal weight b 25 kg/  m^2^ |  | - | NS |  |
|  |  |  |  |  |  |  | Overweight 25–30 kg/m^2^ |  | OR: 1.02 (0.99, 1.06) |  |  |
|  |  |  |  |  |  |  | Obesity N 30 kg/m^2^ |  | OR: 1.00 (0.96, 1.05) |  |  |
| 26 | Potestio (2009) [80] | Proximity | | Average Network to  a park/greenspace (256.2 to 4,639.2 m)) low-moderate- high) | GIS | Child's BMI | Underweight/normal | We used each child's measured height and  weight to calculate their BMI | Distance to Park  (Moderate)  0.94 (0.77-1.16) | NS | Sex and income |
|  |  |  |  |  |  |  | Overweight |  | Distance to Park  (High)  0.88 (0.72-1.07) |  |  |
| 27 | Potwarka (2008) [81] | Proximity | | Locations of parks within the neighborhoods and 800 m buffer  zones | GIS | Child's BMI | _ | Reported by parents | OR: 1.02 (95% CI: 0.73-1.44) | NS | Gender, age, neighborhood of residence, and parent’s BMI |
| 28 | Putrik (2015) [103] | Access/availability | | Quality and availability of greenspace (scale of 0  to 10 satisﬁed) | Questionnaire | BMI | Overweight  (25<BMI≤30 | Self-  reported | OR: 1.08 (95% CI: 0.98, 1.20) | NS | Individual age, gender, and education category |
|  |  |  |  |  |  |  | Obese  BMI>30 |  | OR: 0.84 (95% CI: 0.73, 0.97) |  |  |
| 29 | Rossi (2018) [151] | Proximity (distance to the home (approximately 800 meters) | | 1-10 minutes | to walk the distance  in minutes | BMI/waist circumference | Overweight  for age and sex ≥ +1 and < +2 z-scores | Weight and height data were collected objectively by researchers | OR Lower income: 0.11 (95% CI: 0.07, 0.17) | Negative | Schoolchildren’s age |
|  |  |  |  |  |  |  |  |  | OR High income: 3.03 (95% CI: 0.88, 10.38) | NS |  |
|  |  |  |  | 11 - 19 minutes |  |  | Obesity  for age and sex ≥ + 2 z-scores.  percentile ≥ 90 for age and sex |  | OR Low income: 0.14 (95% CI: 0.03, 0.62) | Negative |  |
|  |  |  |  |  |  |  |  |  | OR High income: 1.07 (95% CI: 0.18, 3.85) | NS |  |
|  |  |  |  | ≥ 20minutes |  |  |  |  | Reference | - |  |
| 30 | Rundle (2013) [65] | Proximity | | Half-mile radius circular  buffers around each subject’s residence | GIS | BMI | Height or weight values or combinations | a BMI of 70 is above the 95  th percentile of  BMI values observed in NHANES | B= −1.69 (95% CI: −2.76, −0.63) | Negative | individual age, gender, race/ethnicity, education and neighborhood percent Black, percent Hispanic, percent poverty and population density |
| 31 | Schüle (2016) [102] | Access/availability | | Greenspace in neighborhood (Lack of greenspace in neighborhood  ) | ArcGIS | Child's BMI | Normal  <25 kg/m2 | Measured by trained staff | OR: 0.94 (95% CI: 0.66, 1.32) | NS | Age, sex, BMI mother, BMI father, Parental education, Contextual neighborhood SEP, age-specific public playground space, and park availability |
|  |  |  |  |  |  |  | Overweight  25 kg/m2-<30 kg/m2 |  |  |  |  |
|  |  |  |  |  |  |  | Obese  >=30 kg/m2 |  |  |  |  |
| 32 | Singh (2010) [66] | Access/availability | | Neighborhood access to parks or playgrounds (yes /No) | Measured by telephone interview | Child's BMI | Overweight  ≥85th percentile | Measured by telephone interview | Access/Prevalence=30.76, SE=0.68  Not Access/Prevalence=34.82, SE=0.19 | NS | Age, sex, race/ethnicity, household composition, metropolitan/nonmetropolitan residence, household poverty or education levels, TV viewing time, recreational computer use, and physical activity |
|  |  |  |  |  |  |  | Obese  ≥95th  percentile |  | Access/Prevalence=15.88, SE=0.56  Not Access/Prevalence=18.27, SE=0.97 |  |  |
| 33 | Sullivan (2014) [67] | Access/availability | | Presence of a park, playground, or open space | Interviewed | BMI | Obesity≥30 kg/m^2^ | Self-reported | OR:  0.68 (95% CI 0.47, 0.98) | Negative | Physical characteristics |
| 34 | Toftager (2011) [104] | Proximity | | Distance to greenspace  <300 m | - | BMI | Obesity BMI> 30 | - | Reference | _ | Sex, age, combined school and vocational education, accommodation type, size of municipality, and long-term activity limitation |
|  |  |  |  | 300 m–1 km |  |  |  |  | OR: 1.01 (95% CI: 0.88-1.17) | NS |  |
|  |  |  |  | >1 km |  |  |  |  | OR: 1.36 (95% CI: 1.08-1.71) | Positive |  |
| 35 | Van der Zwaard (2018) [94] | Access/availability | | Levels  of greenspace, gardens, crime and deprivation (less/ more) | Measures of the amount of greenspace in the local area of these children at each time point | Child's BMI | Overweight vesus normal BMI | (Weight (kg)/height ^2^ (m ^2^ )) | RC: -0.01 (95%CI: -0.032, -0.012) | NS | Age |
| 36 | Veitch (2016) [69] | Australia | Access/availability | Distance to closest park (km) | GIS | BMI | Overweight/obese>=25kg/m^2^ | Self-reported | OR: 0.82 (95% CI: 0.53-1.27) | NS | Age, country of birth/race and household income |
|  |  |  |  | Number of parks in 1600 m buffer |  |  |  |  | OR: 0.98 (95% CI: 0.97-0.99) | Negative |  |
|  |  |  |  | Area of parks in 1600 m buffer (hectares) |  |  |  |  | Reference | - |  |
|  |  | US |  | Distance to closest park (km) |  |  |  |  | OR:1.09 (95% CI: 0.85-1.41) | NS |  |
|  |  |  |  | Number of parks in 1600 m buffer |  |  |  |  | OR: 0.92 (95% CI:0.83-1.01) | NS |  |
|  |  |  |  | Area of parks in 1600 m buffer (hectares) |  |  |  |  | Reference | - |  |
| 37 | Velásquez-Meléndez (2013) [152] | Access/availability | | Presence of parks, squares, and  locations for physical exercise in  coverage area | VIGITEL | BMI | Exceed weight BMI ≥ 25kg/m2 | Self-reported. | PR: 0.88 (95% CI: 0.76-1.03) | NS | Gender, age, conjugal status, and physical inactivity |
| 38 | Veugelers (2008) [82] | Access/availability | | Access to playgrounds and parks (scale of 1 to 5, with 1  representing ‘poor’ and 5 ‘excellent’) | Interviewed | Diet Quality  Index | Sports with a coach/ Sports without a coach / Screen time/ Overweight/ Obesity | parental responses | Overweight  OR (95% CI)  1.01 (0.83;1.24) | children in neighborhoods with good access to playgrounds, parks and recreational facilities were reportedly more active  and were less likely to be overweight or obese, whereas children in safe neighborhoods engaged more in unsupervised sports. |  |
|  |  |  |  |  |  |  |  |  | Obesity  OR (95% CI)  1.04 (0.76;1.44) |  |  |
| 39 | Wall (2012) [70] | Access/availability | | Opportunities for recreational physical activity, including proportion of nearby land used for parks/recreation and distances to the nearest walking/biking trail, recreation center, and  gym/fitness center; | GIS | Adolescent BMI  Z-score | BMI>= 95th percentile | NR | β liner regression  B (SE): Boys: -0.161 (0.066)  P-value=0.014  Girls: -0.129 (0.051)  p-value=0.012 | Negative | Adolescent age, SES, and race/ethnicity |
| 40 | Wen (2012) [71] | Proximity | | Distance to parks (mile) | GIS | BMI | Measured obesity (BMI > 30) | Objectively measured weight and height (kg/m  2) | OR(Men)=  0.90  P-value<0.001 | Negative | Gender, immigrant status, marital status, education, poverty income ratio, and current smoking status, Model 3 added the built environment factors |
|  |  |  |  |  |  |  |  |  | OR (women)=  0.93  P-value<0.001 |  |  |
| 41 | Wolch (2011) [72] | Proximity | | Within a 500 m distance of children’s homes, | GIS | Child's BMI | - | Trained  technician measured kg of  weight/height squared in m. | B Liner regression: -0.1389 (95% CI: -0.0322, -0.2456) | Negative | AADT density, average urban imperviousness, total length of arterial roads, number of ‘‘X’’ intersections, NDVI, and percent below poverty |
|  |  |  |  |  |  |  |  |  | Boys: -0.1863 (95% CI: 0.0267, -0.346) | Negative |  |
|  |  |  |  |  |  |  |  |  | Girls: -0.1034 (95% CI: 0.0354, -0.2423) | NS |  |
| 42 | Yang (2018) [73] | Proximity | | Distance to the nearest park was calculated for  each census block, then aggregated to zip code level; mile | GIS | Child's BMI | Overweight + obesity≥85th percentile | NR | OR:  0.99 (95% CI: 0.99, 1.00)) | NS | Age, gender, race, economically disadvantaged status, school type, and school level |
|  |  |  |  |  |  |  | Obesity ≥95th percentile |  | OR:  0.99 (95% CI: 0.99, 1.00) |  |  |
| 43 | Nesbit (2014) [63] | Access/availability | | Presence of parks and playgrounds (presence / Absence) | GIS | Adolescent BMI | BMI ≥ 95th percentile | respondent (i.e. generally, parent) reported | OR:  0.86 (95% CI: 0.80–0.92) | Negative |  |
| 44 | Pereira (2018) [107] | Proximity | | Older buildings and mix land use | General Directory of  the Territory website | BMI | Obese (BMI: ≥ 30.0) | Trained people | Reference | _ | Urban design clusters, gender, father schooling level, mother's weight status, and father's weight status. |
|  |  |  |  | Small rented buildings and urban areas |  |  |  |  | OR: 0.72 (95% CI: 0.48-1.06) | NS |  |
|  |  |  |  | Newer buildings and urban greenspace |  |  |  |  | OR: 0.48 (95% CI: 0.24-0.97) | Negative |  |
| 45 | Akpinar(2017)[128] | Proximity | | Distance to urban green  spaces by seven levels |  | Children s  Overweight (BMI) | Overweight (Yes/No) | questionnaire  parental responses | The distance from home to urban greenspaces did not have a significant correlation with children's overweight. β (SE):  −0.001 (0.01); p > 0.05. | NS | distance to Urban Greenspace (UGSs) from home/ children`s Physical activity (PA)/ (children`s age, sex, and monthly income)/ children`s PA, screen time, general health, and overweight among  sex and age groups. |
| 46 | Benjamin-Neelon (2019)[75] | green vegetation density | | Define greenspace as  NDVI values ≥ 0.2 and  nongreenspace as <0.2 | NDVI= Normalized Difference Vegetation Index | Body mass index z-score | Body mass index category: Underweight/ Normal weight/ Overweight/ Obese | respondent (i.e. generally, parent) reported | More time spent in greenspace was not associated with a lower BMI z  score in children  in in  Tijuana β (95% CI):  −0.009 (−0.02, 0.004)  or Ensenada β (95% CI):  0.001 (−0.008, 0.01). | NS | Child age, child gender,  family income,  assessment and  accelerometer wear  time. |
| 47 | Bjork (2008)[109] | Access/availability | | Natural recreational  Values  Each geocoded residential address was evaluated for the presence or absence of each of the five recreational values within 100–300 meters of the property's center. | GIS | Overweight/obesity  (BMI) | Grouped as normal (, 25 kg/m2), overweight (25–29 kg/m2) or obese (>30 kg/m2). | questionnaire | The number of recreational values had a positive effect on obesity among tenants (OR (95 %CI): 1.22 (1.06, 1.41) but not among homeowners. | Tenants: Positive | Gender, age, birthplace, educational level, employment status, bill-paying issues, smoking status, and type of residence |
|  |  |  |  |  |  |  |  |  |  | Homeowners |  |
| 48 | Brown(2009)[53] | Access/availability | | Presence of parks (yes or  no) within the 1-km  buffer | - | Overweight/obesity  (BMI), BMI | healthy weight (18.5≤BMI≤24.9), overweight (25≤BMI≤29.9) and obese (BMIZ≥30) | self-reported | The presence of parks within a 1-kilometer street network buffer has no relationship with BMI or overweight/obesity, p > 0.05. | NS | Neighborhood income,  Proportions Black, Hawaiian/Pacific Islander, Hispanic, and Asian, as well as the median age of residents in the neighborhood and individual age. |
| 49 | Browning and  Rigolon (2018)[54] | green vegetation density(greenness) | | Per 0.01-unit increment in  NDVI-250m and  percent tree cover | NDVI | Obesity (BMI) | Obesity=  (BMI) larger  than or equal to 30.0 kg/m2 | self-reported weight and height | The relationship between greenness (per 0.01-unit increment in NDVI-250 m) and obesity was not statistically significant, (SE):0.00037(0.009), p > 0.1. | Greenness  And obesity  NS | Levels of income, race,  and ethnicity and  sprawl |
|  |  |  |  |  |  |  |  |  | Obesity was significantly associated with percent tree cover (SE): 0.026 (0.11), p < 0.05. | percent tree cover  and obesity  Negative |  |
| 50 | Coombes (2010)[96] | Proximity | | Distance to nearest green  space by quartile | GIS | Overweight/obesity  (BMI) | BMI of 25 or above= overweight or obese | questionnaire | In the highest quartile, there was an increase in the odds of being overweight or obese as the distance to the nearest greenspace increased, OR (95% CI): 1.27(1.09,1.47), compared to the lowest quartile. | Positive | Age, sex, socio-economic  status, self-rated health  and area deprivation. |
| 51 | Cummins and  Fagg (2012)[97] | Access/availability | | Percentage of green  space by quintiles | Middle Super Output Areas  (MSOAs) | Overweight/obesity  (BMI) | weight (kg)/height2 (m2)) was categorized using  World Health Organization defifinitions13 of underweight (<18.5 kg m2),  normal (18.5 -- 25 kg m2), overweight (25 -- 30 kg m2) and obese (>30 kg  m2). | Health  Survey for England (HSE) | In the years 2000–2003, living in the greenest areas was associated with an increase in overweight OR (95 %CI): 1.12 (1.03, 1.22) and obesity OR (95% CI): 1.23 (1.11, 1.37) but not in the years 2004–2007. | 2000–2003 year  Positive | Age, gender, social class, economic activity, income deprivation in the neighborhood, and urban/rural status |
|  |  |  |  |  |  |  |  |  |  | 2004–2007  NS |  |
| 52 | Dempsey(2018)[110] | proximity | | Proportion of greenspace  by quintiles | QGIS | Overweight/obesity  (BMI) | BMI≥30 | Interviews | Obesity has a U-shaped relationship with greenspace in  urban areas; those  who live in  areas with the  lowest  and highest shares  of greenspace  Within a 1.6-km buffer zone are more likely to be obese (BMI ≥ 30). | lowest and highest shares  of greenspace  positive | Age, regional location,  gender, income  category, marital status,  employment status,  education level, type of  medical coverage and  Smoking status. |
| 53 | Ellaway  (2005)[98] | Access/availability | | Visible greenery and  vegetation | face to face interview | Overweight/obesity  (BMI) | - | self-reported data on  the health of occupants | In the highest level, greenery was significantly associated with overweight/obesity, with an OR (95 %CI):0.63(0.49, 0.82) compared to the lowest level. | In highest level  Negative | Sex, age, socio-economic  status and city of  residence. |
| 54 | Feng (2018)[140] | Access/availability | | Percentage of land use by  four levels | Statistical Area 2 according to the Australian Bureau of Statistics (ABS) | BMI | - | Australia’s definitive birth cohort, the Longitudinal Study of Australian Children (LSAC) | Compared to mothers in areas with ≤5%greenspace, adjusted BMI coefficients were 0.43 (SE 0.37), 0.69 (SE 0.32), 0.86 (SE 0.33), and 0.80 (SE 0.41) for mothers in areas with 6–10%, 11–20%, 21–40%, and 41% greenspace, respectively. | - | Highest educational  qualification, economic  status and race. |
| 55 | Hoehner  (2012)[56] | Proximity | | Distance to/proportion/  number of parks within  800-m buffer | GIS | BMI | - | Self-reported | There was no significant association between distance to nearest park and BMI, (SE): 0.009 (0.046) p = 0.85; there was no significant association between number of parks and BMI, (SE): 0.012 (0.011) p = 0.24.  There was no significant relationship between the proportion of vegetation and BMI, (SE): 0.562(0.0467) p = 0.23. | NS | Sex, age, marital status,  children in home,  educational status and  Smoking status. |
| 56 | James  (2017)[57] | Proximity | | NDVI-250 m | NDVI | BMI | weight/height^2^ (kg/m^2^) | questionnaire | Increases in greenness were associated with higher BMI at lower greenness levels, but lower BMI at higher greenness levels. | lower greenness levels: Positive | Age, race, and socioeconomic status at the individual and community levels |
|  |  |  |  |  |  |  |  |  |  | higher greenness levels:  Negative |  |
| 57 | Li (2008)[58] | Access/availability | | The total acreage of green  and open spaces | GIS | Overweight/obesity  (BMI) | BMI ≥25= overweight or obese | face-to-face survey interview | The effect estimate of overweight/obesity and green and open spaces was not provided in the article. | NR | Residential density, median household income, percentage of African-American and Hispanic residents, Age, gender, race/ethnicity, employment status, home ownership, household income, health status, fruit and vegetable intake, fried-food consumption, and BMI |
| 58 | Li (2018)[59] | proximity | | GVI | GSV | BMI | individual’s weight (kg) by the square of their height (m) | - | **Females** have a stronger link to lower BMI than males when it comes to street greenery. | Negative mainly in females | Age, gender and residential location of residents |
| 59 | Liu (2007)[60] | proximity | | Per 0.1 increments of  NDVI-2 km | ArcGIS | Overweight (BMI) | obesity =BMI ≥30 kg/m2  overweight =BMI ≥25 kg/m2 | electronic medical record system | In higher population density townships, per 0.1 increment of NDVI-2 km was significantly associated with child overweight, OR (SE): 0.899(1.038), p0.01, but not in low population density townships. | higher population density townships  Negative | Age, race, gender, and median family income in the neighborhood |
|  |  |  |  |  |  |  |  |  |  | low population density townships  NS |  |
| 60 | Mowafi  (2012)[129] | Access/availability | | Number of greenspaces | GIS mapping | BMI | Normal weight (18.0–24.9 kg/m2 )  Overweight (25.0–29.9 kg/m2 )  Obese (≥30 kg/m2 ) | - | There is no link between greenspace and BMI. | NS | Age, marital status, education, household expenditure, household asset index, subjective wealth index, father's education, current general health status, and childhood general health status |
| 61 | Nielsen and  Hansen (2007)[105] | Proximity | | Distance to green areas  (m) | questionnaire | Overweight/obesity  (BMI) | high BMI≥27.5 | questionnaire | Access to a garden or shared greenspace from one's home is associated with a lower risk of obesity, OR = 0.517, p <0.05. | Negative | Employment, level of  education, ownership  to dwelling, age,  gender, household-type  second home and  Bicycling for work. |
| 62 | Norman (2006)[64] | Access/availability | | Number of parks within  1-mile buffer | GIS | BMI | kilograms per square meters/  BMI-for-age percentile | - | There were no statistically significant correlations between environmental variables and BMI percentile for either girls or boys. | NS | NR |
| 63 | Oreskovic  (2009)[49] | Access/availability | | Mean number of parks (in  m2) | GIS | Overweight/obesity  (BMI) | “overweight” as a BMI greater than or equal to the 85th percentile and “obese” as a BMI greater than or equal to the 95th percentile. | Partners HealthCare  Of  Massachusetts | There was no statistically significant relationship between open space and overweight OR (95 %CI): 0.89 (0.92, 1.04), or obesity 0.93. (0.86, 1.00). | NS | Age, gender, race and  income. |
| 64 | Ortega Hinojosa  (2018)[48] | Access/availability | | NDVI-1 km | NDVI  Geological Survey | BMI | above the 95th percentile | FITNESSGRAM | The most highly ranked built or physical environment variables were distance to the nearest highway and greenness, but they were not selected or included in the models. | NR | Age, gender, year and  race. |
| 65 | Pearson  (2014)[142] | Access/availability | | Proportion of useable  greenspace by five  levels 1 = best access and 5 = worst access | - | Overweight/obesity  (BMI) | 1) overweight; 2) obesity; 3) overweight + obesity | Interviews | Overweight status was associated with a lower proportion of greenspace in each category when compared to the best access, OR (95 %CI): 1.39. (1.10, 1.75). | negative | Age, sex, ethnicity,  Economic Living  Standard Index,  individual-level  deprivation, highest  educational  qualification, household  composition, smoking  status and alcohol  Consumption. |
| 66 | Pereira  (2013)[143] | proximity | | NDVI-1.6 km (low,  moderate and high  level) | NDVI | Overweight/obesity  (BMI) | ***Obese* i**f their BMI (weight (kg)/height (meters) ^2^) exceeded 30, and as ***overweight-or-obese***if the BMI was above 25. | the Health and Wellbeing Survey | Adults in neighborhoods with high levels of mean greenness (highest tertile) had a lower adjusted odds ratio for being overweight/obese, OR (95 %CI): 0.84 (0.76, 0.92), than those in neighborhoods with low levels of mean greenness. | Negative mainly in adults in neighborhoods with high levels of mean greenness | Age, sex, education, daily  servings of fruits and  vegetables and  Smoking. |
| 67 | Li(2022)[180] | Proximity | | remote sensing inversion on the basis of medium resolution satellite images, with a spatial resolution of 250 m  (NDVI) | ArcGIS | BMI | BMI > 28 kg/m2 as the definition of obesity | questionnaire data | the protective effect of greenspace on obesity in NDVI with 500 m and 1000 m buffer  after adjusting for the same factors for greenspace, found a 49.8% reduction in the risk of obesity for each 0.1 increase in NDVI (OR: 0.502, 95% CI: 0.336, 0.751) | Negative | age, gender, marital status, education level, career, family income, daily sitting time, weekly outdoor exercise frequency, type of house, house area, straight-line distance between home and road, residents' evaluation of ambient air pollution, smoking, drinking, personal or family history of chronic noncommunicable diseases. |
| Abbreviations: POS: public open space; BMI, body mass index; GPS: Global Positioning System; Km: Kilometer; Kg: Kilogram; GIS: Geographical Information System; NDVI: normalized difference vegetation index; NS: Not significant; NR: Not reported; RC: Regression coefficient; SE; Standard error; MRC: Multilevel regression coefficient: PR: prevalence ratios; SC: Spearman correlation; U.S.: United States; CI: Confidence Interval; GSV: Google Street View; GVI: green view index | | | | | | | | | | | |

| Supplementary Table 2: Main finding of included studies about the association between greenspace exposure and HTN | | | | | | | | | | |
| --- | --- | --- | --- | --- | --- | --- | --- | --- | --- | --- |
| NO. | Author (Year) | POS Characteristics | | | Outcome | | | Quantitative | Finding | Description |
|  |  | Type | definition | measurement | Type | definition | measurement |  |  |  |
| 68 | Abbasi (2020)  [15] | proximity | As parks, land covered with growing trees, agricultural ﬁelds, gardens, etc15-minute walk from their home | Questionnaire | SBP | levels that are ≥90th  percentile | Sphygmomanometer  twice following a  5-minute interval, and the average was recorded | 95% conﬁdence intervals (CIs):  −0.58, 0.41, p value= 0.72) | Positive | Residential proximity to greenspaces was associated with 0.08mmHg reduction in SBP |
|  |  |  |  |  | DBP | levels that are  less than  95th  percentile |  | −0.09 (95%  CIs: −0.49, 0.31, p value= 0.66) | Positive | Residential proximity to greenspaces was associated with 0.09 mmHg reduction in DBP |
| 69 | Thomas Astell-Burt (2016)[139] | proximity | 1 km proximity bands as the most basic definition of ‘exposure’ | GIS | health outcome | BMI and incidence of doctor-diagnosed cardiometabolic diseases such as hypertension, cardio-vascular disease and diabetes | self-reported | 0.9 (0.8 to 1.1) | Negative | There were virtually no significant differences in the distribution of any of health outcomes |
| 70 | Regina Grazuleviciene(2014)[89] | proximity | three greenspace distance categories to the nearest park, using circular buffers with a radius of <300 m, 300–1,000 m, and >1,000 m | ArcGIS | blood pressure | high-normal blood pressure (SBP 130 to 139 mm Hg or DBP 85 to 89 mm Hg) | well-trained physician using a mercury column  sphygmomanometer placed on the right arm,  The mean value of two blood pressure over a two-minute interval in a seated position, after five minutes of rest were reported | crude ORs 1.12, 95% CI 0.82–1.52 (moderate distance) and 1.83, 95% CI 1.22–2.75, (farthest distance) | Positive | There was statistically significant association between increasing distance to city parks, and increase in the odds ratios for high-normal blood pressure |
|  |  |  |  |  |  | hypertension (SBD ≥ 140 or DBP ≥ 90 mm Hg) |  | crude ORs 1.00, 95% CI 0.77–1.33 (moderate distance) and 1.18, 95% CI 0.81–1.72(farthest distance) | Negative | There was no statistically significant association between the proximity of city parks and the hypertension group |
| 71 | Sérgio Rodrigues Moreira (2013)[150] | Park users | Park users | - | SBP | SBP≥140 mmHg | automatic BP monitor on the left arm after resting for 10 min while sitting in a chair | OR  1.17  95% CI  (0.41 –3.34) | Negative | There was no significant association between physical activity levels and SBP |
|  |  |  |  |  | DBP | DBP≥90mmHg |  | OR  0.68  95% CI  (0.16 –2.80) | Negative | There was no significant association between physical activity levels and DBP |
| 72 | Iana Markevych(2014) [181] | greenness | 500-m buffers around current home addresses of participants | ArcGIS 10.0 and GME | SBP | SBP | BP measured by two physicians between 7:00 a.m.  and 8:30 p.m. using an automatic BP monitor | p-value = 0.073 (low greenness), 0.014  (Moderate greenness) | Positive | The SBP of children living at residences with low and moderate greenness was 0.90 ± 0.50 mmHg and 1.23 ± 0.50 mmHg higher, respectively, than the SBP of children living in areas of high greenness. |
|  |  |  |  |  | DBP | DBP |  | p-value = 0.033 (low greenness), 0.011 (moderate greenness) | Positive | The DBP of children living in areas  with low and moderate greenness was 0.80 ± 0.38 mmHg and 0.96 ± 0.38 mmHg  higher, than the DBP of children living in areas of high greenness. |
| 73 | Usama Bilal(2016)[182] | urban environment | measuring specific characteristics of urban environments in Spain | GIS | cardio-vascular risk | This cardio-vascular risk assessment includes the measurement of blood pressure and lipids and assessing tobacco use | using Electronic Health Records and qualitative interviews | - | Negative | 34 % of population had a diagnosis of hypertension |
| 74 | H SusanJ Picavet(2016) (73) | greenness | percentage of green within 1 kilometer radius and within 125m radius | several versions of the National Land Cover Classification Database | Health includes: CVD (myocardial infarction, coronary heart diseases, and intermittent claudication  ), Blood pressure | SBP | Mean of two measured BP or questionnaires | β  0.13  (-0.20; 0.46) | Positive | Greener within a 1km radius was associated with higher systolic blood pressure |
|  |  |  |  |  |  | Hypertension:  SBP ≥ 130 mmHg, or DBP ≥ 85 mmHg, or use of  antihypertensive medication |  | agricultural green  OR  0.99  (0.95; 1.02)  Urban green OR  1.02  (0.96; 1.08) | Positive | Based on type of green: more agricultural green was associated with higher blood pressure and more urban green with a lower blood pressure |
| 75 | Bo-Yi Yang (2019)[14] | greenness | NDVI  SAVI | ArcGIS 10.4 | SBP | SBP | BP were measured by trained nurses using the standard mercuric-column  sphygmomanometer method in a sitting position,  after a minimum of five-minute rest, and>30 min after exercising,  tobacco smoking, and consuming tea, coffee, or alcohol | NDVI500-m  (95% CI: −1.13, −0.51)  SAVI500-m  (95% CI: −1.21, −0.57) | Negative | An interquartile  range increase in both NDVI500-m and SAVI500-m were associated with 0.082 mmHg and 0.89 mmHg reduction in SBP respectively. |
|  |  |  |  |  | hypertension | Hypertension:  SBP≥140 mmHg or  DBP≥90 mmHg, or reported use of anti-hypertensive medicine |  | NDVI500-m  (95% CI: 1%, 8%)  SAVI500-m  (95% CI: 1%, 9%) | Positive | An interquartile  range increase in both NDVI500-m and SAVI500-m were significantly associated with 5% and 5% lower odds of having hypertension, respectively. |
| 76 | Scott C. Brown(2016)[26] | greenness | mean NDVI for each resident’s Census block | - | Chronic  health conditions including hypertension, hyperlipidemia, diabetes | standardized protocol | Data were obtained for this retrospective cohort study in 2013  from the United States CMS  Master Beneﬁciary Summary File | OR  (95% CI)  0.927  (0.911,  0.943)  p-value  <0.0001 | Negative | Each 0.1-unit increase in NDVI is associated with a significantly reduction (7%) in risk of hypertension |
| 77 | Catherine Paquet(2014) [141] | POS size | deﬁned as  parcels larger than a typical urban house block (700 m^2^) used as  sporting facilities, reserves, national parks, conservation reserves,  or botanic gardens. | ArcGIS | cardio-metabolic risk factors (pre-diabetes/ diabetes, hypertension, dyslipidemia, abdominal obesity) | diastolic/systolic BP ≥ 85/  130 mmHg or treatment for hypertension | obtained during clinic visits | RR 95% CI  1.08 (0.95,1.24) | Negative | There was no association between POS and hypertension. |
|  |  | POS greenness |  |  |  |  |  | RR 95% CI  0.97 (0.87,1.07) |  |  |
|  |  | POS type |  |  |  |  |  | RR 95% CI  0.92 (0.80,1.06) |  |  |
| 78 | Angel M. Dzhambov(2018)[16] | proxy  for accessibility greenspace | distance to  urban greenspaces ≤300 m, 301–500-m, and>500-m | ArcGIS | SBP | based on the first and  fifth Korotkoff phase using a fixed deflation rate of 3mm | Before and after interview using a calibrated mercury  sphygmomanometer with a large scale on the right arm in a sitting position after a three-minute rest | β=−3.36; 95% CI: −6.24, −0.48 | Negative | NDVI 500-m was associated with lower  SBP. |
|  |  |  |  |  | DBP |  |  | β=−1.17; 95% CI: −3.10, 0.75 | NS | NDVI 500-m was not significantly associated with lower  DBP. |
| 79 | Esmée M Bijnens(2017)[12] | Greenness | Greenness 5000 m buffer  1000 m buffer  300m buffer | GIS | SBP | SBP was measured. SBP>220 mmHg or <70 mmHg was automatically rejected. | Ambulatory blood pressure was monitored using the  Spacelabs 90,207 device at home on the nondominant  arm. Recording began between 6.00 and 9.00 AM and  was finished 24 h later. The recorders  were taken every  15 min during daytime (8.00 AM to 10.00 PM) and every  30 min during night-time (10.00 PM to 8.00 AM). | 95% CI: -6.0 to −1.23;  p = 0.005 | Negative | An interquartile  increase in residential greenness exposure within 1000 m residential radius was associated with a decrease of  3.59 mmHg in night-time SBP |
|  |  |  |  |  | DBP | DBP was measured. DBP>140 mmHg or <40 mmHg was automatically rejected. |  | 95% CI: -4.2 to −0.6;  p = 0.01 | Negative | Diastolic blood pressure during  the night was significantly associated with residential  greenness in a 300 m buffer |
| 80 | Jie Jiang(2020)[13] | long-term greenness | Defined as the average of NDVI and EVI values in 500-m buﬀer around each home | NDVI & EVI | SBP | SBP ≥ 140 mm Hg or DBP ≥ 90 mm Hg or they had taken antihypertensive medicine or had been diagnosed with hypertension by a professional clinician | Blood pressure was measured three times with a three-minute interval on the right arm of participants in  a sitting position after a five-minute rest. Participants were not allowed to smoke, drink alcohol, coffee  or tea, and exercise at least 30 minutes before the measurement.  Average value of the three-blood pressure reported. | 95% CI:  -1·17, - 0·58 | Negative | An IQR increase in NDVI 500m was significantly reduced 0.88 mmHg of SBP |
|  |  |  |  |  | DBP |  |  | 95% CI:  - 0·82, - 0·46 | Negative | An interquartile range (IQR) increase in NDVI 500m was significantly reduced 0.64 mmHg of DBP |
| 81 | Marcia P. Jimenez(2020)[17] | Proximity | distance (miles) to the closest  greenspace  one mile  , the average area (hectares) of greenspace within the  neighborhood, and number of greenspaces (n) within the neighborhood (or greenspace count) | GIS | SBP | Mean  of the second and third measured blood pressure was reported. | In Ed Health participants: five SBP and  DBP measures were obtained over 1-min intervals in participants seated,  after 5 min rest, in the right arm at heart level, using automated  blood pressure monitors  In LEAP participants: three BP measures  were assessed by certified research nurses using mercury sphygmomanometers  in seated participants resting 5 min prior to assessment | 5.6  95%CI: 0.7, 10.5 | Positive | Living one  mile farther from a greenspace at birth was associated with an increase  of 5.6 mmHg in SBP |
|  |  |  |  |  | DBP |  |  | 1.5  95%CI:  0.3, 6.8 | Positive | Living one mile farther from a park at birth was associated with an increase of  1.5 mmHg in DBP |
| 82 | Ray Yeager(2018)[47] | Residential Greenness | NDVI  Residential NDVI values were divided into high (>0.55), medium (0.36–0.54), and low (<0.36) categories based on tertile of contemporaneous NDVI values within a circular zone of 250 m around the participant's residence | ArcMap9.3+  GIS | Cardiovascular disease risk (Hypertension, Hyperlipidemia, Diabetes mellitus, Current smoker, High CVD risk) | SBP | Extract from cohort study | Low Green  131.8    Medium Green  129.7  High Green  131.4  P Value:  0.710 | Negative | There was no significant association between increasing greenspace and SBP |
|  |  |  |  |  |  | DBP |  | Low Green  80.1  Medium Green  80.8  High Green  81.3  P Value:  0.747 | Negative | There was no significant association between increasing greenspace and DBP |
| 83 | Li (2022)[131] | Proximity | The normalized differential vegetation index (NDVI) of Anhui Province in 2020 was produced and processed by remote sensing inversion on the basis of medium resolution satellite images. | geocoded residential address | HTN | Chinese Guidelines for Prevention and Treatment of HTN | - | The adjusted odd ratio of HTN for each 1 μg/m3 increasing in 0.1 of NDVI was 0.669 (0.611, 0.733). | Negative | - |
| 84 | Aliyas et al. (2018)[137] | Access | Parks are located within a 10-minute walk from their homes on a 4-point scale | Questionnaire | HTN | Hypertension was deﬁned as  SBP ≥ 140 mmHg, and/or DBP ≥ 90 mmHg,  and/or when the respondent had been taking  drugs for high BP during the last two weeks | trained students after 5 minutes of participants ’ rest | Odds Ratio (95% CI)  0.47(0.30,0.79) | Positive | Access to parks positively was correlated with perceived better physical health (bp); however,  results indicated no association with mental health. |
| 85 | Bauwelinck et al. (2020)[126] | Proximity | (1) NDVI and MSAVI2 (buffers of  100 m, 300 m, and 500 m);(2) Total surface of green spaces  (m2) for buffers of 300 m and  500 m around the residential  address.;  (3)Residential distance to  nearest green space | GIS | HTN | Self-reported hypertension by combining  it with self-reported use of hypertension medication | Self-reporting of doctor-diagnosed | Barcelona  Adjusted OR (95% CI) 1.15  (1.03 – 1.29) | Positive | We observed a protective association between residential distance to nearest green space and risk of hypertension, especially in older age. |
|  |  |  |  |  |  |  |  | Brussels  Adjusted  OR (95% CI) 0.95  (0.77 – 1.17) |  |  |
| 86 | Bloemsma et al. (2019)[125] | Residential  greenness | (1) NDVI (buffers of  300 m,3000 m);  (2) Total percentage of green  space;  (3) Agricultural and natural  green space in circular buffers of  300 m and 3000 m around the adolescents' homes | GIS | SBP | NA | SBP and DBP were measured using an Omron M6 monitor  ac-  cording to the recommendations of the American Heart Association  Council on High Blood Pressure Research | β (95% CI)  0.13 (−0.86, 1.12)  β (95% CI) 0.20 (−0.50, 0.90) | Negative | We did not observe consistent patterns of associations of greenspace , airpollution and traffic noise with  the cardiometabolic risk score, blood pressure, total cholesterol levels |
|  |  |  |  |  | DBP |  |  |  |  |  |
|  |  |  |  |  |  |  |  |  |  |  |
| 87 | Braziene et al. (2019)[124] | Proximity | Distance to greenspace:  ≤ 150 m | ArcGIS | HTN | Hypertension was deﬁned as SBP ≥ 140 mmHg  and/or a DBP ≥ 90 mmHg, or self-reporting of  medication prescribed for hypertension during  the preceding two weeks | SBP  > 120 mmHg  DBP  ≥ 80 mmHg  *Medians of corresponding variables* | Reference  RR (95% CI)=1 | Positive | An increase in the incidence of arterial hypertension was associated with a shorter distance to  a major road and a greater distance to a city park. |
|  |  |  | 151–300 m |  |  |  |  | RR (95% CI)=1.49 (1.03–2.15) |  |  |
|  |  |  | > 300 m |  |  |  |  | RR (95% CI)=1.51 (1.10–2.07) |  |  |
| 88 | de Keijzer et al. (2019)[123] | Proximity | NDVI (buffer of 500 m) | Satellite-based | HTN | Hypertension was defined as SBP  ≥140 mmHg and/or DBP ≥90 mmHg, or  use of antihypertensive medication | The Hawksley random-zero  sphygmomanometer | Hazard ratios (95% confidence interval)  0.88 (0.79, 0.97) | Negative | Greater exposure to greenspace was also associated with each individual component of  metabolic syndrome, including a lower risk of high levels of fasting glucose, large waist circumference,  high TG levels, low HDL cholesterol, and HTN. |
|  |  |  | VCF (buffer of 500 m) |  |  |  |  | Hazard ratios (95% confidence interval)  0.84 (0.77, 0.93) |  |  |
|  |  |  |  |  |  |  |  |  |  |  |
| 89 | Jendrossek et al. (2017)[21] | Residential greenness | NDVI (buffer of 500 m) | Landsat 5 Thematic Mapper (TM) satellite images | HTN | NA | Self-report of doctor-diagnosed hypertension | Munich OR (95% CI)=1.101 (0.718 to 1.688) Wesel OR (95% CI)=1.065 (0.671 to 1.691) | NS | No significant and consistent associations across different levels of adjustment were observed between the exposures of interest and HTN. |
| 90 | Leng et al. (2020)[136] | Residential greenness | Per IQR (0.17) increment in  NDVI or NDVI (buffers of 250 m  and 1000 m) | Satellite-derived | HTN | SBP | Automated oscillometer BP device | 4:3mmHg(95%CI=2.9,5.6) | Negative | Residing in areas  With lower greenness, was significantly associated with elevated SBP,DBP, and cPP, adjusting for age,BMI, sex, smoking status, and other CVD risk factors. |
|  |  |  |  |  |  | DBP |  | 1:2mmHg(95%CI=0.4,2.0) |  |  |
|  |  |  |  |  |  | cPP |  | 3:1mmHg(95%CI=2.0,4.1) |  |  |
| 91 | Madhloum et al. (2019)[122] | Residential greenness | An IQR (20.3%) increment in  percentage residential greenness  in a 5 km radius | GIS | HTN | SBP | Standardized protocol | 95%CI =−1.2 (−2.5; 0.1) | Negative | An IQR (20.3%) increment in percentage residential  greenness in a 5 km radius was associated with a 1.2mmHg lower SBP and a  1.2mmHg lower DBP |
|  |  |  |  |  |  | DBP |  | 95%CI =−1.2 (−2.4;  −0.0) |  |  |
| 92 | Plans et al. (2019)[121] | Access | Per IQR increase density of green  spaces (buffers of 300 m, 500 m,  1000 m and 1500 m) | GIS | HTN | Hypertension was defined as  SBP ≥ 140 mmHg or DBP ≥ 90 mmHg, or  previously diagnosed with hypertension | Electronic health records | OR(CI 95%)  =0.98 (0.71–1.37) | Negative | Moderate association between density of green spaces  and HTN, high cholesterol, and diabetes,  but not for obesity; particularly, females living in areas of lower green space density had greater odds  for specific cardiovascular risk factors compared to  those that live in the highest density areas (Q1). |
| 93 | Poulsen et al. (2021)[88] | Proximity | (1) An interquartile range  increase in NDVI (buffer of  1250 m);  (2) Percent Forest within  1250 m square buffers | GIS | SBP | NA | Measurement per  individual from an outpatient primary care visit during the second year after meeting the  criteria for Type2 diabetes  diagnosis. | Townships Beta (95% CI)=-0.87 (-1.43, -0.30) | Negative | In townships, the greenest  communities,an interquartile range increase in NDVI was associated with reductions in SBP of  0.87 mmHg (95% CI: -1.43, -0.30) and in DBP of 0.41 mmHg (95% CI: -0.78, -0.05). |
|  |  |  |  |  | DBP |  |  | Townships Beta (95% CI)=-0.41 (-0.78, -0.05) |  |  |
| 94 | Ribeiro et al. (2019)[120] | Access | (1) Green space at 400 m/800 m  from school/residence (Yes or No)  (2) Distance to the nearest greenspace | GIS | allostatic load (SBP/  DBP) | allostatic load - based on seven biomarkers  representing four regulatory systems: immune/inflammatory system (high sensitivity C-reactive protein(CRP)); metabolic  system (high density lipoprotein; total cholesterol; glycated hemoglobin; waist-hip ratio) and cardiovascular  system (systolic and diastolic blood pressure). | NA | Beta (95% CI)=  −0.19(−0.50 to 0.12)/  Beta( 95% CI)=0.05  (−0.06 to 0.15) | Negative | Analyses of individual  biomarkers also showed associations between presence of a  green space near school (Yes/No) and cardiovascular biomarkers (SBP  and DBP) and inflammation measured by hsCRP. |
| 95 | Riggs et al. (2021)[87] | Residential Greenness | Per 0.1 in SD with a 200 m/1 km  buffer increase in NDVI | satellite | SBP | NA | Self-reported and medical records | -14.7 mmHg (-28.1 to -1.3) | Negative | For a 0.1 increase in SD within a 200-mradius, we found negative  associations with augmentation index (_7.4%; 95% CI:  _13.5, _1.3), augmentation pressure (_4.8mmHg; 95% CI:  _9.0, _0.7), and aortic systolic pressure (_14.7mmHg; 95%  CI: _28.1, _1.3). |
| 96 | Sarkar et al. (2018)[119] | Residential Greenness | Per IQR increase NDVI (buffer of  500 m) | GIS | HTN | Hypertension was defined as SBP≥140 mmHg,  and/or DBP≥90 mmHg, or self-reported use of  antihypertensive medication | Measured at baseline using  standardized procedures by trained nurses | β (95% CI) =0.970  (0 | Negative | Each interquartile increment in walkability  was associated with the lower blood pressure outcomes of DBP (β=−0.358, 95% CI: −0.42, −0.29 mmHg),  SBP (β=−0.833, 95% CI: −0.95, −0.72 mmHg) as well as reduced HTN risk (RR=0.970, 95% CI:  0.96, 0.98). |
|  |  |  |  |  | SBP |  |  | β (95% CI)  =−0.833 (−0.95,−0.72) |  |  |
|  |  |  |  |  | DBP |  |  | β (95% CI)  =−0.358 (−0.42,−0.29) |  |  |
| 97 | Tamosiunas et al. (2014)[118] | Access | Per tertile distance from people’s  residence to green spaces (living  farther away than 347.81 m) | GIS | HTN | Defined as mean SBP  of at least 140 mm Hg or mean DBP of at least  90 mm Hg, or both, and/or when the respondent had  been taking drugs for high BP during the last two weeks. | Mercury sphygmomanometer | OR (95% CI)=0.92 (0.81-1.04) | NS | No  significant relationship was found between the distance  to green spaces and the prevalence of arterial hypertension. |
| 98 | Ulmer et al. (2016)[86] | Access | Park percentage within 500 m  buffer and Tree cover percentage  within 250 m buffer. | GIS | HTN | NA | Self-reporting of doctor-diagnosed | RR (95% CI)=1.113 (1.094,1.132) | Negative | The results indicated that more neighborhood tree cover, independent from green space access, was related to better overall health, primarily mediated by lower overweight/obesity and better social cohesion, and to a lesser extent by less type 2 diabetes, high blood pressure, and asthma. |
| Abbreviations: POS: Public open space, SBP, Systolic blood pressure; DBP, Diastolic blood pressure; CI: Confidence Interval; km: Kilometer; mmHg: millimeter of mercury; GIS: geographic information system; OR: Odds ratio; BP: Blood pressure; GME: Geospatial  Modelling Environment; CVD: Cardiovascular disease; NDVI: normalized difference vegetation index; SDVI: Soil Adjusted Vegetation Index; CMS: Centers for Medicare and Medicaid Services; EVI: Enhanced Vegetation Index; IQR: interquartile range, cPP=central pulse pressure. | | | | | | | | | | |

| Supplementary Table 3: Main finding of included studies about the association between greenspace exposure and and Diabetes | | | | | | | | | | |
| --- | --- | --- | --- | --- | --- | --- | --- | --- | --- | --- |
| 99 | Authors (Year) | POS Characteristics | | | Outcome | | | Finding | | |
|  |  | Type | Definitions | Measurement | Type | Definitions | Measurement | Quantitative | Qualitative | Covariate adjustments |
|  | Ruijia Li (2021)[132] | Residential Green and Blue Spaces | Normalized difference  vegetation index NDVI within a 500 m buffer radius  Enhanced  & Vegetation Index (EVI) | Arc GIS 10.2 | type 2 diabetes  mellitus (T2DM) | (1) defects in insulin secretion or action of the participants were not caused by type  1 diabetes mellitus and gestational diabetes mellitus; (2) the patient had been diagnosed Toxics 2021, 9, 11 4 of 13  with T2DM or had been taking antidiabetic drugs directed by doctors; and (3) the fasting  blood glucose level exceeded 7.0 mmol/L. | A Roche Cobas c501 (Switzerland, Basel) analyzer | OR (95%CI)  0.880 (0.837,0.926) | Negative | age (years) and sex (male, female). Additionally,  health status covariates were assessed, including body mass index (BMI) (kg/m  2  ) and  family history of diabetes mellitus (no, yes). The following socioeconomic covariates were examined: marital status (married/living together, divorced/widowed/separated/unmarried),  education level (no school or primary school, middle school, junior college or higher), and  monthly income level (low, medium, or high). We also tested the following health behavior  covariates: smoking status (never, former, or current), drinking status (never, former, or  current), high-fat diet (average consumptions of meat from livestock and poultry by each  participant of more than 75 g per day) (no, yes), fruit and vegetable intake (average intake  of fruits and vegetables by each participant of more than 500 g per day) (no, yes), and  physical activity (low, medium, or high) |
|  |  |  |  |  |  |  |  | OR (95%CI) 0.875 (0.825,0.929) |  |  |
|  |  |  |  |  | fasting blood glucose (FBG) | the fasting  blood glucose level exceeded 7.0 mmol/L.in age >65 |  | %Change  (95%CI) −1.268  (−1.614,−0.921) |  |  |
|  |  |  |  |  |  |  |  | %Change  (95%CI) −1.109  (−1.513,−0.702) |  |  |
| 100 | Annie Doubleday (2022) [84] | Residential Greenspace  (greenness) | NDVI  within  500 m and 2.5 km, as well as high vegetation season (April  1 – September 30) median NDVI within a 1 km radius. | satellite  imagery | Type 2 diabetes | defined as having a fasting glucose level of at least  126 mg/dL at the exam, use of insulin, or use of hypo-  glycemic medication. | clinical visit | HR (95% CI) 0.83 (0.66, 1.03) | Negative | adjustment set: age, sex, race/ethnicity, education category, income  category, employment status, neighborhood deprivation index, neighborhood  social cohesion, neighborhood walkability, neighborhood safety, urbanicity, and  site |
| 101 | LucíaRodriguez-Loureiro(2022)[183] | residential greenness | objective (surrounding greenness) NDVI 500-m and 1000-m |  | diabetes mortality | using the ICD-10 codes E10-E14, including both type 1 (insulin-dependent) and type 2 (non-insulin-dependent) DM. | death certificates with any mention of DM | HR 1.02 (95%CI:0.99,1.06) | Positive | age, gender, household living arrangement (cohabiting with a partner, single, or other), and migrant background [Belgian, other high-income country (HIC), and low and middle-income countries (LMIC)] to assess the individual sociodemographic characteristics |
|  |  |  | subjective (perceived neighbourhood greenness) percentage of households reporting the provision of  neighbourhood greenspaces as “very well equipped” |  |  |  |  | HR 0.93 (95%CI:0.91,0.95) |  |  |
| 102 | Catherine Paquet (2014)[141] | greenness | NDVI  deﬁned as  parcels larger than a typical urban house block (700 m  2  ) Used as  sporting facilities, reserves, national parks, conservation reserves,  or botanic gardens. | ArcGIS | pre-diabetes/ diabetes | HbA1c Z5.7% or fasting plasma glucose Z5.6 mmol/L or  diagnosed diabetes | clinic visits | RR (95% CI )  1.03 (0.93,1.14) | Positive |  |
|  |  |  |  |  |  |  |  | RR (95% CI )  1.01 (0.90,1.13) |  |  |
| 103 | Jiaqiang Liao(2019)[133] | greenness | mean value of the normalized difference vegetation index( NDVI  ) within a 300-meter circular buffer area surrounding each residence | - | Maternal Glucose | Measured between 24 and 28 weeks of gestation and gestational IGT and GDM | visit | IGT (RR: 0.92, 95% CI: 0.86, 0.99 | Negative | residence area (rural, 217  urban), maternal age ( ≤ 24, 25-35, ≥ 36 years), maternal education ( ≤ 9, 10-12, ≥ 13 218  years), maternal household income (< 30000, 30000-50000, 50000-1000000, ≥ 219  100000 yuan), maternal prepregnancy BMI (underweight: <18.5, normal: 18.5-24, 220  overweight or obesity: 24 kg/m  2  ), passive smoking during pregnancy (yes, no), 221  maternal parity (1: nulliparous women, ≥ 2: parous women), and season of conception 222  (spring, summer, autumn, and winter). |
|  |  |  |  |  |  |  |  | GDM (RR: 0.85, 95% CI: 0.79, 0.92) |  |  |
| 104 | AnnaPonjoan(2022) [114] | greenness | NDVI | - | Diabetes | myocardial infarction with diabetes | visit | HR = 0.94; 95%CI, 0.89–0.99 | association between greenness and incidence of myocardial infarction was significant only in men | adjusted by demographic and clinical characteristics at individual level, and by environmental and socioeconomic variables at census tract level |
| 105 | Roland Ngom(2016)[154] | Accessibility | 0.00–264.59 m | ArcGIS  Postal code | Diabetes (types 1 and 2) | use diagnostic codes from the International Classiﬁcation of Diseases | Health databases from surveillance | Prevalence Rate Ratio 95% CI  - | Positive | Age , gender |
|  |  |  | 264.60–468.62 m |  |  |  |  | 1.04( 0.99–1.08) |  |  |
|  |  |  | 468.63–774.42 m |  |  |  |  | 1.02( 0.98–1.07) |  |  |
|  |  |  | 774.43–27,781.92 m) |  |  |  |  | 1.09( 1.03–1.13) |  |  |
| 106 | Soumya Mazumdar(2021)[146] | Access | green space within 500 m, 1 km, and 2 km circular buffers,  LBRN buffers and PBRN buffers around participants’ residences were used as proxies  for geographic access to greenspace | GIS- Postal code | Prevalence of T2D | determine a diagnosis of T2D in the survey were “Has  a doctor ever told you that you have diabetes?” and “Have you taken Diabex, Diaformin,  Metformin for most of the last 4 weeks?” | self-reported | OR 95% CI  1 | Negative | age, gender, BMI, marital status, educational attainment high school and less than 10 years of schooling, ancestry and employment status |
|  |  |  |  |  |  |  |  | 0.72 0.64–0.82 |  |  |
|  |  |  |  |  |  |  |  | 0.72 0.64–0.80 |  |  |
|  |  |  |  |  |  |  |  | 0.55 0.49–0.62 |  |  |
|  |  |  |  |  |  |  |  | 0.39 0.34–0.45 |  |  |
| 107 | Charlotte Clark(2017)[85] | Residential greenness | 100-m buﬀers around residential postal code | measured using the satellite-derived  Normalized Diﬀerence Vegetation Index (NDVI) | diabetes | Standardized Canadian deﬁnitions for identifying diabetes | self-reporting | Adj.OR (95% CI)  0.90 (0.87, 0.92) | Negative | age and gender |
|  |  | Walkability | around residential postal codes that may in ﬂ uence opportunities  for physical activity |  |  |  |  | Adj.OR (95% CI)  1.01 (0.98, 1.04) |  |  |
| 108 | Danielle H Bodicoat(2014)[115] | Percentage of greenspace( greenness) | as the area within 800 m (approximating  to a 10 min walk) of a home location.  Quartiles were de ﬁ ned as ≤ 30%,  31 – 59%, 60 – 77% and ≥ 78% based on the data | ArcGIS | Type 2 diabetes | gold-standard oral glucose tolerance tests (fasting  glucose ≥ 7.0 mmol/L or 2 h glucose ≥ 11.1 mmol/L) or  glycated hemoglobin (HbA1c; ≥ 6.5%; 48 mmol/mol) | Leicester Practice Risk Score | Adjusted † OR (95% CI)  0.96 (0.73 to 1.27) | Negative | ethnicity, age, sex, area social deprivation score, and urban/rural status |
|  |  |  |  |  |  |  |  | 0.71 (0.54 to 0.93) |  |  |
|  |  |  |  |  |  |  |  | 0.65 (0.50 to 0.85) |  |  |
| 109 | Alice M. Dalton(2016)[116] | greenness | defined as the percentage of the home  neighbourhood that was woodland, grassland, arable land, mountain, heath and bog, according to the UK Land  Cover Map | GIS | incident diabetes | uses  data from the baseline survey | using mul-  tiple data sources, including self-report of doctor-diagnosed  diabetes from the second health check or follow-up health  and lifestyle questionnaires, self-report of diabetes-specific  medication in either of the two follow-up questionnaires or  medication brought to the follow-up health check (as de-  scribed in detail elsewhere | Adjusted for confounders 95% CI HR95% CI 1 | Negative | age, BMI, sex, parental diabetes and SES |
|  |  |  |  |  |  |  |  | HR95% CI 0.97 ( 0.80 -1.18) |  |  |
|  |  |  |  |  |  |  |  | HR95% CI 0.83 ( 0.67 -1.02) |  |  |
|  |  |  |  |  |  |  |  | HR95% CI 0.81 ( 0.65- 0.99) |  |  |
| 110 | Shanley Chong,(2019)[145] | percentage of greenspace(greenness) | within 500 m, 1 km  and 2 km polygon-based road network (PBRN) buffers  around participants’ residences | ArcGIS | T2D | defined as those participants who  did not report T2D at the baseline survey but reported  T2D at the follow-up survey | Self-reported | - | NS | age, gender, country of birth  (English-speaking countries, Europe, Middle-East, Asia Other)) and an area-level deprivation score |
| 111 | Grit Müller(2018)[101] | accessibility of green  space | minimum distance from the participants’ resi-  dential addresses to the closest park or forest planting and forests | GIS | T2D | based on the self-reported physi-  cian diagnosis of diabetes | The HbA1c is a measure of glycemic control  and has been established as a screening biomarker for  type 2 diabetes | OR (95%CI)  1.91 (1.20 to 3.04) | Positive | age and sex. |
| 112 | Thomas Astell-Burt(2014)[184] | Proximity | a 1-km buffer from a participant ’ s place  of residence.  1) 0 – 20, 2) 21 – 40, 3)  41 – 60, 4) 61 – 80, and 5) . 80% green  space. | ArcGIS | type 2 diabetes mellitus (T2DM) | - | Doctor diagnosed | OR (95% CI)0.98 (0.95 – 1.02) | Negative | adjusted for age-group, sex, couple status, ancestry, country of birth, and language spoken  at home. |
| 113 | [H.Lee](https://www.sciencedirect.com/science/article/pii/S0033350615002383?via%3Dihub#!)(2015)[185] | Proximity | Area of parks in neighbourhood including 1-km buffer (km^2^) | GIS | diabetes | exhibited a fasting glucose level ≥126 mg/dl | Medical record | Adjusted OR (95% CI) 0.86 (0.75–0.99) | Negative | for age, sex, smoking status, drinking status and income level. |
| 114 | [PayamDadvand](https://www.sciencedirect.com/science/article/abs/pii/S0269749118320992?via%3Dihub#!) (2018)[35] | Greenspace use | average hours per week spent in green  spaces | - | Fasting Blood Glucose | IFG, FBG≥110 mg/dL) base on WHO | enzymatically by a Hitachi 917 automated biochemistry analyzer (Roche Diagnostics,  Indianapolis, IN). | Regression coefficient (95% CIs) -0.5 (-0.9, -0.1) | Negative | age, sex, low birth weight, diet, parental and family history of diabetes mellitus, parental obesity, urbanity, parental educational attainment, home ownership,  and marital status. |

| Supplementary Table 4 : Main finding of included studies about the association between greenspace exposure and lipid profiles | | | | | | | | | | |
| --- | --- | --- | --- | --- | --- | --- | --- | --- | --- | --- |
|  | Authors (Year) | POS Characteristics | | | Outcome | | | Finding | | |
|  |  | Type | Definitions | Measurement | Type | Definitions | Measurement | Quantitative | Qualitative | Covariate adjustments |
|  | Shujun Fan (2020)[23] | Residential greenness | buﬀers of 100 m, 300 m, 500 m, and 1000 m around each participant’ residential address centroid. | ArcGIS 10.4 | TC | hypercholesterolemia as TC  levels of at least 6.22 mmol/L, hypertriglyceridemia as TG levels of at  least 2.26 mmol/L, hypoalphalipoproteinemia as HDL-C levels of <  1.04 mmol/L, and hyperbetalipoproteinemia as LDL-C levels of at least  4.14 mmol/L | using a Roche Autoanalyzer (Cobas c702 type; Roche  Ltd.; Mannheim, Germany) in Guangzhou Center for Disease Control  and Prevention | β (95% CI)  −0.004 (−0.05, 0.04) | Negative | Living in greener areas was associated with lower TG levels, higher HDL-C levels, and lower odds of  hypoalphalipoproteinemia (e.g., per 0.20-unit increase in NDVI 100 m was associated with 0.02 mmol/L (95% CI:  0.001, 0.03) higher levels of HDL-C and 0.87-fold (95% CI: 0.77, 0.98) lower odds of hypoalphalipoproteinemia) |
|  |  |  |  |  | LnTG |  |  | β (95% CI)  −0.01 (−0.03, 0.003) |  |  |
|  |  |  |  |  | HDL-C |  |  | β (95% CI)  0.02 (0.001, 0.03) |  |  |
|  |  |  |  |  | LDL-C |  |  | β (95% CI)  −0.02 (−0.06, 0.03) |  |  |
| 116 | Scott C. Brown(2016)[26] | level of  greenness | vegetative presence | Mean NDVI was derived for  all Miami-Dade County Census blocks for 2011 | hyperlipidemia | PROC GLIMMIX was used to model  the relationship of NDVI to health in a multilevel framework | identiﬁed for each Medicare beneﬁciary for the calendar year 2011, using the 2011 CMS Master Beneﬁciary Summary File | OR (95% CI)  0.941  (0.924,0.959) | Negative | increase in mean Normalized Difference Vegetation Index was associated with a reduced risk  of diabetes by 14%, hypertension by 13%, and hyperlipidemia by 10%. Planned post-hoc analyses  revealed stronger and more consistently positive relationships between greenness and health in  lower- than higher-income neighborhoods. |
| 117 | Hye-Jin Kim (2016)[27] | Greenness | the number of parks and green areas (m  2  ) per capita in 200 administrative districts: Quartile 1 (≤14.90 m  2  /capita), Quartile 2  (14.90–22.40 m  2  /capita), Quartile 3 (22.41–33.30 m  2  /capita), and Quartile 4 (≥33.31 m  2  /capita) | geographical codes | Hyperlipidemia, physician diagnose | included the existence and treatment of hyperlipidemia (when participants  had a history of hyperlipidemia) using self-reported questionnaires | hyperlipidemia-related questions | Adjusted  OR (95%CI)  1.32 (1.25–1.40) | Positive | age, sex, stress,  a history of diabetes, body mass index (BMI), and physical activity |
|  |  |  |  |  | Hyperlipidemia, current treatment |  |  | Adjusted  OR (95%CI)  1.52 (1.40–1.64) |  |  |
| 118 | Hari S. Iyer(2020)[134] | neighborhood greenness | NDVI  Values range from 1 to + 1, | Google Earth Engine,  geocodes | Cholesterol (continuous) | defining high cholesterol as any  value above 6.22 mmol/L based on clinical guidelines used in 2011 | measurements taken by trained study staff | β (95% CI)  0.04 ( - 0.07, 0.14) | Negative | Covariate : age, BMI, systolic blood pressure, diastolic blood pressure, blood  glucose level |
|  |  |  |  |  | Cholesterol (binary) |  |  | OR (95% CI)  1.15 (0.93, 1.42) |  |  |
| 119 | Jie Jiang(2021)[117] | Greenness | a 500-m/1000-m/3000-m buffer according to the  geocoded home address | NDVI, EVI | dyslipidemia | as hypercholesterolemia (TC ≥ 6.2 mmol/L), hypertriglyceridemia (TG ≥ 2.3 mmol/L),  hyperbetalipoproteinemia (LDL-C ≥ 4.1 mmol/L), or  hypoalphalipoproteinemia (HDL-C <1.0 mmol/L). | trained laboratorians in the medical center in the local com-  munity | OR (95% CI) adjusted 1.01 (0.96,1.07) | Positive for dyslipidemia | age, sex, matrimony, educational level, and  monthly income in the associations between NDVI 1000-m and  dyslipidemia and lipid levels |
|  |  |  |  |  | hypercholesterolemia |  |  | 1.05 (0.96,1.14) |  |  |
|  |  |  |  |  | hypertriglyceridemia |  |  | 0.97 (0.91,1.03) |  |  |
|  |  |  |  |  | hyperbetalipoproteinemia |  |  | 1.33(1.21,1.46) |  |  |
|  |  |  |  |  | hypoalphalipoproteinemia |  |  | 0.94(0.88,1.00) |  |  |
| 120 | Iana Markevych(2016)[135] | residential  greenness | NDVI in 100-m, 300- and 500-m buffers around residences | GIS | total cholesterol, | according to the manufactures instructions  (Roche Diagnostics GmbH Mannheim) | by homogenous enzymatic col-  orimetric methods according to the manufactures instructions  (Roche Diagnostics GmbH Mannheim) | MR (95% CI) 1.00 (0.99 – 1.01) | NS | There is no evidence of an association between greenness and blood lipids in 10- and 15-  years old children. |
|  |  |  |  |  | HDL |  |  | ß7SE 0.0170.01 |  |  |
|  |  |  |  |  | LDL |  |  | MR (95% CI) 1.00 (0.99 – 1.01) |  |  |
|  |  |  |  |  | triglyceride |  |  | MR (95% CI) 0.98 (0.96 – 1.01) |  |  |
| 121 | Catherine Paquet(2014)[141] | Greenness | as  parcels larger than a typical urban house block (700 m  2  ) used as  sporting facilities, reserves, national parks, conservation reserves,  or botanic gardens | ArcGIS | dyslipidemia | (triglycerides Z1.7 mmol/L or high-density lipoprotein (HDL) o1.03  (males)/o1.29(females), or treatment with lipid-modifying medication) | - | Adjusted RR ( 95% CI) 1.12 (1.00,1.25) | Positive | adjusted for age, gender, education, household income and area-level deprivation |
| 122 | Bo-Yi Yang(2019)[153] | Residential greenness | NDVI (100-500-1000 m)  SAVI(100-500-1000) | ArcGIS 10.4 | total cholesterol, | hypercholesterolemia as TC >= 240 mg/dL; hyper-  triglyceridemia as TG >= 200 mg/dL; hypoalphalipoproteinemia as  HDL-C <=40 mg/dL; and hyperbetalipoproteinemia as LDL-C  >=160 mg/dL | measured on a  Hitachi Autoanalyzer | OR=1.52 (95% CI)- (-1.80, -1.22) | Negative | Adjusted for age, sex, ethnicity, education level, household income, and district-level gross domestic product |
|  |  |  |  |  | HDL |  |  | OR=0.52 (0.20, 0.85) |  |  |
|  |  |  |  |  | LDL |  |  | -1.91 (-2.44, -1.38) |  |  |
|  |  |  |  |  | triglyceride |  |  | -3.05 (-3.82, -2.28) |  |  |
